# Optimization Techniques for Design Problems in Selected Areas in WSNs: A Tutorial

**DOI:** 10.3390/s17081761

**Published:** 2017-08-01

**Authors:** Ahmed Ibrahim, Attahiru Alfa

**Affiliations:** 1 Faculty of Engineering and Applied Sciences, Memorial University of Newfoundland, St. John’s, NL A1B 3X5, Canada; 2 Department of Electrical and Computer Engineering, University of Manitoba, Winnipeg, MB R3T 5V6, Canada; Attahiru.Alfa@umanitoba.ca; 3 Department of Electrical, Electronic and Computer Engineering, University of Pretoria, Pretoria 0002, South Africa

**Keywords:** wireless sensor networks, design, optimization, tutorial

## Abstract

This paper is intended to serve as an overview of, and mostly a tutorial to illustrate, the optimization techniques used in several different key design aspects that have been considered in the literature of wireless sensor networks (WSNs). It targets the researchers who are new to the mathematical optimization tool, and wish to apply it to WSN design problems. We hence divide the paper into two main parts. One part is dedicated to introduce optimization theory and an overview on some of its techniques that could be helpful in design problem in WSNs. In the second part, we present a number of design aspects that we came across in the WSN literature in which mathematical optimization methods have been used in the design. For each design aspect, a key paper is selected, and for each we explain the formulation techniques and the solution methods implemented. We also provide in-depth analyses and assessments of the problem formulations, the corresponding solution techniques and experimental procedures in some of these papers. The analyses and assessments, which are provided in the form of comments, are meant to reflect the points that we believe should be taken into account when using optimization as a tool for design purposes.

 **Contents**
 

**I** **Introduction****4****II****Overview on Mathematical Optimization****7****1****The History of Optimization****8****2****Introduction to Mathematical Programming****9****3****Linear Programming Problems****9**
3.1 Solving LPs . . . . . . . . . . . . . . . . . . . . . . . . . . . . . . . . . . . . . . . . . . . . . . . . . . . . . . . . . . . . . . . . . . . . . . . . . . . . . . . . . . . . . . . . . .10
  3.1.1 Simplex Method . . . . . . . . . . . . . . . . . . . . . . . . . . . . . . . . . . . . . . . . . . . . . . . . . . . . . . . . . . . . . . . . . . . . . . . . . . . . . . . . .10
  3.1.2 Karmarkar’s Algorithm . . . . . . . . . . . . . . . . . . . . . . . . . . . . . . . . . . . . . . . . . . . . . . . . . . . . . . . . . . . . . . . . . . . . . . . . . . .11
  3.1.3 On the Efficiency of Simplex and Karmarkar’s Algorithms . . . . . . . . . . . . . . . . . . . . . . . . . . . . . . . . . . . . . . . . . . . . .12
3.2 Linear Duality . . . . . . . . . . . . . . . . . . . . . . . . . . . . . . . . . . . . . . . . . . . . . . . . . . . . . . . . . . . . . . . . . . . . . . . . . . . . . . . . . . . . . . . .12
  3.2.1 The Dual Simplex . . . . . . . . . . . . . . . . . . . . . . . . . . . . . . . . . . . . . . . . . . . . . . . . . . . . . . . . . . . . . . . . . . . . . . . . . . . . . . . .13**4****Network Flow Programming Models****13**
4.1 Terminology . . . . . . . . . . . . . . . . . . . . . . . . . . . . . . . . . . . . . . . . . . . . . . . . . . . . . . . . . . . . . . . . . . . . . . . . . . . . . . . . . . . . . . . . .15
4.2 Special Classes of NFPs . . . . . . . . . . . . . . . . . . . . . . . . . . . . . . . . . . . . . . . . . . . . . . . . . . . . . . . . . . . . . . . . . . . . . . . . . . . . . . . .16
  4.2.1 Transportation Problem . . . . . . . . . . . . . . . . . . . . . . . . . . . . . . . . . . . . . . . . . . . . . . . . . . . . . . . . . . . . . . . . . . . . . . . . . .16
  4.2.2 Assignment Problem . . . . . . . . . . . . . . . . . . . . . . . . . . . . . . . . . . . . . . . . . . . . . . . . . . . . . . . . . . . . . . . . . . . . . . . . . . . . .17
  4.2.3 Transshipment Problem . . . . . . . . . . . . . . . . . . . . . . . . . . . . . . . . . . . . . . . . . . . . . . . . . . . . . . . . . . . . . . . . . . . . . . . . . .18
  4.2.4 Shortest Path Problems . . . . . . . . . . . . . . . . . . . . . . . . . . . . . . . . . . . . . . . . . . . . . . . . . . . . . . . . . . . . . . . . . . . . . . . . . . .19
  4.2.5 Maximum Flow Problems . . . . . . . . . . . . . . . . . . . . . . . . . . . . . . . . . . . . . . . . . . . . . . . . . . . . . . . . . . . . . . . . . . . . . . . . .20
  4.2.6 Minimum Spanning Tree (MST) . . . . . . . . . . . . . . . . . . . . . . . . . . . . . . . . . . . . . . . . . . . . . . . . . . . . . . . . . . . . . . . . . . . .21
  4.2.7 Minimum Cost Network Flow Problems . . . . . . . . . . . . . . . . . . . . . . . . . . . . . . . . . . . . . . . . . . . . . . . . . . . . . . . . . . . . .21**5****Fundamentals of Nonlinear Optimization****22**
5.1 Lagrange Functions and Multipliers . . . . . . . . . . . . . . . . . . . . . . . . . . . . . . . . . . . . . . . . . . . . . . . . . . . . . . . . . . . . . . . . . . . . .25
5.2 Karush-Kuhn-Tucker (KKT) Conditions . . . . . . . . . . . . . . . . . . . . . . . . . . . . . . . . . . . . . . . . . . . . . . . . . . . . . . . . . . . . . . . . . .25
  5.2.1 Geometric Interpretation of KKT Necessary Conditions . . . . . . . . . . . . . . . . . . . . . . . . . . . . . . . . . . . . . . . . . . . . . . . .26
5.3 Duality for Nonlinear Programs . . . . . . . . . . . . . . . . . . . . . . . . . . . . . . . . . . . . . . . . . . . . . . . . . . . . . . . . . . . . . . . . . . . . . . . . .26**6****Decomposition Methods****27**
6.1 Primal Decomposition . . . . . . . . . . . . . . . . . . . . . . . . . . . . . . . . . . . . . . . . . . . . . . . . . . . . . . . . . . . . . . . . . . . . . . . . . . . . . . . . .27
6.2 Dual Decomposition . . . . . . . . . . . . . . . . . . . . . . . . . . . . . . . . . . . . . . . . . . . . . . . . . . . . . . . . . . . . . . . . . . . . . . . . . . . . . . . . . . .28**III****Optimization in WSN Design Problems****29****7****Routing for Multi-hop WSNs with a Single Immobile Sink****32**
7.1 System Model and Design Objectives . . . . . . . . . . . . . . . . . . . . . . . . . . . . . . . . . . . . . . . . . . . . . . . . . . . . . . . . . . . . . . . . . . . .32
7.2 Initial Optimization Problem Formulation . . . . . . . . . . . . . . . . . . . . . . . . . . . . . . . . . . . . . . . . . . . . . . . . . . . . . . . . . . . . . . . .32
7.3 Reformulation . . . . . . . . . . . . . . . . . . . . . . . . . . . . . . . . . . . . . . . . . . . . . . . . . . . . . . . . . . . . . . . . . . . . . . . . . . . . . . . . . . . . . . . .32
7.4 Solution Methods . . . . . . . . . . . . . . . . . . . . . . . . . . . . . . . . . . . . . . . . . . . . . . . . . . . . . . . . . . . . . . . . . . . . . . . . . . . . . . . . . . . . .32
  7.4.1 A partially Distributed Algorithm . . . . . . . . . . . . . . . . . . . . . . . . . . . . . . . . . . . . . . . . . . . . . . . . . . . . . . . . . . . . . . . . . .32
  7.4.2 A fully Distributed Algorithm . . . . . . . . . . . . . . . . . . . . . . . . . . . . . . . . . . . . . . . . . . . . . . . . . . . . . . . . . . . . . . . . . . . . .33
7.5 Important Results . . . . . . . . . . . . . . . . . . . . . . . . . . . . . . . . . . . . . . . . . . . . . . . . . . . . . . . . . . . . . . . . . . . . . . . . . . . . . . . . . . . . .33
7.6 Discussions of the Results . . . . . . . . . . . . . . . . . . . . . . . . . . . . . . . . . . . . . . . . . . . . . . . . . . . . . . . . . . . . . . . . . . . . . . . . . . . . . .34**8****Routing in a delay-tolerant WSN to a Single Mobile Sink****34**
8.1 System Model and Design Objectives . . . . . . . . . . . . . . . . . . . . . . . . . . . . . . . . . . . . . . . . . . . . . . . . . . . . . . . . . . . . . . . . . . . .34
8.2 Initial Optimization Problem Formulation . . . . . . . . . . . . . . . . . . . . . . . . . . . . . . . . . . . . . . . . . . . . . . . . . . . . . . . . . . . . . . . .34
8.3 Comments on the Problem Formulation . . . . . . . . . . . . . . . . . . . . . . . . . . . . . . . . . . . . . . . . . . . . . . . . . . . . . . . . . . . . . . . . . .35
8.4 Reformulation for the Optimization Problem Formulation . . . . . . . . . . . . . . . . . . . . . . . . . . . . . . . . . . . . . . . . . . . . . . . . . .35
8.5 Solution Method . . . . . . . . . . . . . . . . . . . . . . . . . . . . . . . . . . . . . . . . . . . . . . . . . . . . . . . . . . . . . . . . . . . . . . . . . . . . . . . . . . . . .35
8.6 Important Results . . . . . . . . . . . . . . . . . . . . . . . . . . . . . . . . . . . . . . . . . . . . . . . . . . . . . . . . . . . . . . . . . . . . . . . . . . . . . . . . . . . .36**9****Joint Routing, Power and Bandwidth Allocation in FDMA WSNs****36**
9.1 System Model and Design Objectives . . . . . . . . . . . . . . . . . . . . . . . . . . . . . . . . . . . . . . . . . . . . . . . . . . . . . . . . . . . . . . . . . . .36
9.2 Optimization Problem Formulation . . . . . . . . . . . . . . . . . . . . . . . . . . . . . . . . . . . . . . . . . . . . . . . . . . . . . . . . . . . . . . . . . . . . .36
9.3 Reformulation: Distributed Global Consensus Problem . . . . . . . . . . . . . . . . . . . . . . . . . . . . . . . . . . . . . . . . . . . . . . . . . . . .37
9.4 Solution Method . . . . . . . . . . . . . . . . . . . . . . . . . . . . . . . . . . . . . . . . . . . . . . . . . . . . . . . . . . . . . . . . . . . . . . . . . . . . . . . . . . . . .37
9.5 A Reference Algorithm . . . . . . . . . . . . . . . . . . . . . . . . . . . . . . . . . . . . . . . . . . . . . . . . . . . . . . . . . . . . . . . . . . . . . . . . . . . . . . .38
9.6 The Obtained Results . . . . . . . . . . . . . . . . . . . . . . . . . . . . . . . . . . . . . . . . . . . . . . . . . . . . . . . . . . . . . . . . . . . . . . . . . . . . . . . . .38**10****Routing and Energy Allocation in Rechargeable WSNs with Multiple Sources and Destinations****39**
10.1 System Model and Design Objectives and Considerations . . . . . . . . . . . . . . . . . . . . . . . . . . . . . . . . . . . . . . . . . . . . . . . . .39
10.2 Problem Formulation . . . . . . . . . . . . . . . . . . . . . . . . . . . . . . . . . . . . . . . . . . . . . . . . . . . . . . . . . . . . . . . . . . . . . . . . . . . . . . . .39
10.3 Comments on the Problem Formulation . . . . . . . . . . . . . . . . . . . . . . . . . . . . . . . . . . . . . . . . . . . . . . . . . . . . . . . . . . . . . . . .40
10.4 Solution Method . . . . . . . . . . . . . . . . . . . . . . . . . . . . . . . . . . . . . . . . . . . . . . . . . . . . . . . . . . . . . . . . . . . . . . . . . . . . . . . . . . . .40
10.5 Comments and Observations on the Solution Technique . . . . . . . . . . . . . . . . . . . . . . . . . . . . . . . . . . . . . . . . . . . . . . . . . .41
10.6 Comments on the Experiments and Results . . . . . . . . . . . . . . . . . . . . . . . . . . . . . . . . . . . . . . . . . . . . . . . . . . . . . . . . . . . . .41**11****Routing in Multi-hop Single Immobile Sink for Different Objectives under Distance Uncertainties****42**
11.1 Problem Statement and Design Objectives . . . . . . . . . . . . . . . . . . . . . . . . . . . . . . . . . . . . . . . . . . . . . . . . . . . . . . . . . . . . . . .42
11.2 Formulations for the Three Problems . . . . . . . . . . . . . . . . . . . . . . . . . . . . . . . . . . . . . . . . . . . . . . . . . . . . . . . . . . . . . . . . . . .42
11.3 Accounting for Uncertainties in the Formulation . . . . . . . . . . . . . . . . . . . . . . . . . . . . . . . . . . . . . . . . . . . . . . . . . . . . . . . . .43
11.4 Important Results . . . . . . . . . . . . . . . . . . . . . . . . . . . . . . . . . . . . . . . . . . . . . . . . . . . . . . . . . . . . . . . . . . . . . . . . . . . . . . . . . . .44**12****Joint Routing and Scheduling in WSNs with Multiple Sinks having Different Location Possibilities****45**
12.1 System Model and Design Objectives . . . . . . . . . . . . . . . . . . . . . . . . . . . . . . . . . . . . . . . . . . . . . . . . . . . . . . . . . . . . . . . . . .45
12.2 Initial Problem Formulation: Time Based Formulation . . . . . . . . . . . . . . . . . . . . . . . . . . . . . . . . . . . . . . . . . . . . . . . . . . . .45
12.3 Reformulation: Pattern Based Formulation . . . . . . . . . . . . . . . . . . . . . . . . . . . . . . . . . . . . . . . . . . . . . . . . . . . . . . . . . . . . . .46
12.4 Solution Method: Column Generation Method . . . . . . . . . . . . . . . . . . . . . . . . . . . . . . . . . . . . . . . . . . . . . . . . . . . . . . . . . . .46
12.5 Important Results . . . . . . . . . . . . . . . . . . . . . . . . . . . . . . . . . . . . . . . . . . . . . . . . . . . . . . . . . . . . . . . . . . . . . . . . . . . . . . . . . . .47**13****Delay-Sensitive Routing in Underwater WSNs****47**
13.1 System Model and Design Objectives . . . . . . . . . . . . . . . . . . . . . . . . . . . . . . . . . . . . . . . . . . . . . . . . . . . . . . . . . . . . . . . . . .47
13.2 Initial Problem Formulation . . . . . . . . . . . . . . . . . . . . . . . . . . . . . . . . . . . . . . . . . . . . . . . . . . . . . . . . . . . . . . . . . . . . . . . . . .47
13.3 Comments on the Initial Problem Formulation . . . . . . . . . . . . . . . . . . . . . . . . . . . . . . . . . . . . . . . . . . . . . . . . . . . . . . . . . .48
13.4 Reformulation . . . . . . . . . . . . . . . . . . . . . . . . . . . . . . . . . . . . . . . . . . . . . . . . . . . . . . . . . . . . . . . . . . . . . . . . . . . . . . . . . . . . . .48
13.5 Comments on Reformulation . . . . . . . . . . . . . . . . . . . . . . . . . . . . . . . . . . . . . . . . . . . . . . . . . . . . . . . . . . . . . . . . . . . . . . . . .48
13.6 The Solution Method in and Our Comments on it . . . . . . . . . . . . . . . . . . . . . . . . . . . . . . . . . . . . . . . . . . . . . . . . . . . . . . . .48**14****Using Mobile Radio Frequency (RF) Power Charger to Charge the Batteries of Sensors in a WSN****49**
14.1 System Model and Design Objectives . . . . . . . . . . . . . . . . . . . . . . . . . . . . . . . . . . . . . . . . . . . . . . . . . . . . . . . . . . . . . . . . . . .49
14.2 Problem Formulation . . . . . . . . . . . . . . . . . . . . . . . . . . . . . . . . . . . . . . . . . . . . . . . . . . . . . . . . . . . . . . . . . . . . . . . . . . . . . . . .49
14.3 Solution Method . . . . . . . . . . . . . . . . . . . . . . . . . . . . . . . . . . . . . . . . . . . . . . . . . . . . . . . . . . . . . . . . . . . . . . . . . . . . . . . . . . . .50
14.4 Observations and Comments on the Problem Formulation, Solution Method and Experiments . . . . . . . . . . . . . . . . . .50**15****Assignment of Processing Tasks Across Nodes in a WSN****50**
15.1 System Model and Design Objectives . . . . . . . . . . . . . . . . . . . . . . . . . . . . . . . . . . . . . . . . . . . . . . . . . . . . . . . . . . . . . . . . . .50
15.2 Initial Optimization Problem Formulation . . . . . . . . . . . . . . . . . . . . . . . . . . . . . . . . . . . . . . . . . . . . . . . . . . . . . . . . . . . . . .51
15.3 Solution Method . . . . . . . . . . . . . . . . . . . . . . . . . . . . . . . . . . . . . . . . . . . . . . . . . . . . . . . . . . . . . . . . . . . . . . . . . . . . . . . . . . .51
15.4 Comments on the Solution Method . . . . . . . . . . . . . . . . . . . . . . . . . . . . . . . . . . . . . . . . . . . . . . . . . . . . . . . . . . . . . . . . . . . .51**16****Hierarchical Clustering in a Heterogeneous Network****51**
16.1 System Model and Design Objectives . . . . . . . . . . . . . . . . . . . . . . . . . . . . . . . . . . . . . . . . . . . . . . . . . . . . . . . . . . . . . . . . . . .51
16.2 Problem Formulation . . . . . . . . . . . . . . . . . . . . . . . . . . . . . . . . . . . . . . . . . . . . . . . . . . . . . . . . . . . . . . . . . . . . . . . . . . . . . . . .52
16.3 Solution Method . . . . . . . . . . . . . . . . . . . . . . . . . . . . . . . . . . . . . . . . . . . . . . . . . . . . . . . . . . . . . . . . . . . . . . . . . . . . . . . . . . . .52
16.4 Comments on the Solution Method . . . . . . . . . . . . . . . . . . . . . . . . . . . . . . . . . . . . . . . . . . . . . . . . . . . . . . . . . . . . . . . . . . . .52**17****Energy Efficient Co-Operative Broadcasting at the Symbol Level****52**
17.1 System Model and Design Objectives . . . . . . . . . . . . . . . . . . . . . . . . . . . . . . . . . . . . . . . . . . . . . . . . . . . . . . . . . . . . . . . . . .52
17.2 Optimization Problem Formulation . . . . . . . . . . . . . . . . . . . . . . . . . . . . . . . . . . . . . . . . . . . . . . . . . . . . . . . . . . . . . . . . . . . .53
17.3 Comments on the Optimization Problem Formulation . . . . . . . . . . . . . . . . . . . . . . . . . . . . . . . . . . . . . . . . . . . . . . . . . . . .53
17.4 Optimal Solution Method and Our Related Comments . . . . . . . . . . . . . . . . . . . . . . . . . . . . . . . . . . . . . . . . . . . . . . . . . . . .53
17.5 Suboptimal Solution Method: A Heuristic Technique . . . . . . . . . . . . . . . . . . . . . . . . . . . . . . . . . . . . . . . . . . . . . . . . . . . . .54
17.6 Important Numerical Results . . . . . . . . . . . . . . . . . . . . . . . . . . . . . . . . . . . . . . . . . . . . . . . . . . . . . . . . . . . . . . . . . . . . . . . . .54
17.7 Comments on the Solution Methods and Their Related Experiments . . . . . . . . . . . . . . . . . . . . . . . . . . . . . . . . . . . . . . . .54**18****Dispatching of Mobile Sensor Nodes in a WSN to Sense a Region of Interest****54**
18.1 System Model and Design Objectives . . . . . . . . . . . . . . . . . . . . . . . . . . . . . . . . . . . . . . . . . . . . . . . . . . . . . . . . . . . . . . . . . . .54
18.2 Problem Formulation . . . . . . . . . . . . . . . . . . . . . . . . . . . . . . . . . . . . . . . . . . . . . . . . . . . . . . . . . . . . . . . . . . . . . . . . . . . . . . . .55
18.3 Solution Method: Centralized . . . . . . . . . . . . . . . . . . . . . . . . . . . . . . . . . . . . . . . . . . . . . . . . . . . . . . . . . . . . . . . . . . . . . . . . .55
18.4 Solution Method: Distributed . . . . . . . . . . . . . . . . . . . . . . . . . . . . . . . . . . . . . . . . . . . . . . . . . . . . . . . . . . . . . . . . . . . . . . . . .55
18.5 Comments on the Solution Methods . . . . . . . . . . . . . . . . . . . . . . . . . . . . . . . . . . . . . . . . . . . . . . . . . . . . . . . . . . . . . . . . . . .56**19****Fusion of Delay Sensitive Noise Perturbed Data Sensed by Different Nodes in a Given Cluster****56**
19.1 System Model and Design Objectives . . . . . . . . . . . . . . . . . . . . . . . . . . . . . . . . . . . . . . . . . . . . . . . . . . . . . . . . . . . . . . . . . . .56
19.2 Optimization Problem Formulation . . . . . . . . . . . . . . . . . . . . . . . . . . . . . . . . . . . . . . . . . . . . . . . . . . . . . . . . . . . . . . . . . . . .57
19.3 Comments on the Formulation . . . . . . . . . . . . . . . . . . . . . . . . . . . . . . . . . . . . . . . . . . . . . . . . . . . . . . . . . . . . . . . . . . . . . . . .57
19.4 Solution Method: Heuristic scheme . . . . . . . . . . . . . . . . . . . . . . . . . . . . . . . . . . . . . . . . . . . . . . . . . . . . . . . . . . . . . . . . . . . .58
19.5 Comments on the Solution Methods . . . . . . . . . . . . . . . . . . . . . . . . . . . . . . . . . . . . . . . . . . . . . . . . . . . . . . . . . . . . . . . . . . .58**20****Energy Optimization in Wireless Visual Sensor Networks While Maintaining Image Quality****58**
20.1 System Model . . . . . . . . . . . . . . . . . . . . . . . . . . . . . . . . . . . . . . . . . . . . . . . . . . . . . . . . . . . . . . . . . . . . . . . . . . . . . . . . . . . . . .58
20.2 Formulation . . . . . . . . . . . . . . . . . . . . . . . . . . . . . . . . . . . . . . . . . . . . . . . . . . . . . . . . . . . . . . . . . . . . . . . . . . . . . . . . . . . . . . . .59
20.3 Solution . . . . . . . . . . . . . . . . . . . . . . . . . . . . . . . . . . . . . . . . . . . . . . . . . . . . . . . . . . . . . . . . . . . . . . . . . . . . . . . . . . . . . . . . . . . .60**21****Conclusions****60**

## Part I Introduction

As the technology evolves, the wireless sensors manufactured become technically more powerful and economically viable. In wireless sensor networks (WSN) each node consists basically of units for sensing, processing, radio transmission, position finding and sometimes mobilizers [1]. These sensors measure desired phenomenal conditions in their surroundings and digitize them to process the received signals to reveal some characteristics of the conditions in the surrounding area. A large number of these sensors can be networked in many applications that require unattended operations, hence producing a WSN. In general, the sensor nodes in a wireless sensor network WSN sense and gather data from surrounding environment and transmit it to one or more sinks, to perform more intensive processing. The number of applications for WSNs is large, many of these are in the fields of weather monitoring, surveillance, health care, detecting ambient conditions like temperature, sound, light, security related aspects, etc. More fields are deploying WSNs as their reliability, performance and capabilities keep getting even better and wider.

In many applications, replacement of damaged or energy depleted nodes is not possible. Moreover planned nodal placement may not be a possible thing to do. Therefore, two of the main requirements for WSNs to operate reliably are to consume the minimum amount of energy to prolong the network’s life time, and to be able to self organize themselves when the network topology changes. Other requirements (e.g., limited delay, good signal to noise ratio, etc.) are usually application specific. Moreover, there are differences in the nature of WSNs. For example, there could be WSNs with either rechargeable or non-rechargeable sensor batteries, either single sink WSNs or multiple sink WSNs, which could either be immobile or mobile. Depending on these different variants of WSNs, different types of applications and the traffic types they handle, different design considerations will need to be taken into account. An acoustic WSN, for example, would need to consider propagation delays in any design aspect which most likely will be ignored in radio based WSNs. Also, as another example if voice or sound is to be sensed and transmitted for surveillance applications, then strict delay requirements for routing the packets will have to be enforced.

Besides the application types and the WSN variants, there are many aspects such as routing, clustering, accurately estimating sensed data, visual target tracking etc. in which optimal design is needed. In all these design problems, energy is a common limitation for the reasons mentioned earlier. For this paper we selected key optimization aspects of design problems relating to WSNs, carried out a thorough review of the selected major papers and used that opportunity to present a tutorial on the subject.

The papers selected for this review and tutorial are based on how well they covered the topics of interest and the degree to which they highlight the key design issues for WSN. In part III of this paper, we expose the reader to the following design problems in WSNs:

1. routing for multi-hop WSNs with a single immobile sink [2],
2. routing in a delay-tolerant WSN with a single mobile sink [3],
3. joint routing, power and bandwidth allocation in FDMA WSNs [4],
4. joint energy allocation and routing in WSNs with rechargeable batteries [5],
5. routing in multi-hop single fixed sink with different objectives under distance uncertainties [6],
6. joint routing and scheduling in WSNs with multiple sinks with different sink location possibilities [7],
7. delay sensitive routing for underwater WSNs [8],
8. using mobile radio frequency (RF) power charger to charge the batteries of sensors in a WSN [9],
9. assignment of data processing tasks across the nodes in WSN [10],
10. hierarchical clustering in a heterogeneous network [11],
11. energy efficient co-operative broadcasting at the symbol level [12],
12. dispatching of mobile sensor nodes in a WSN to sense a particular region of interest [13],
13. fusion of delay sensitive noise perturbed data sensed by different nodes in a given cluster [14],
14. Energy Optimization in Wireless Visual Sensor Networks While Maintaining Image Quality [15].

**Figure 1:**
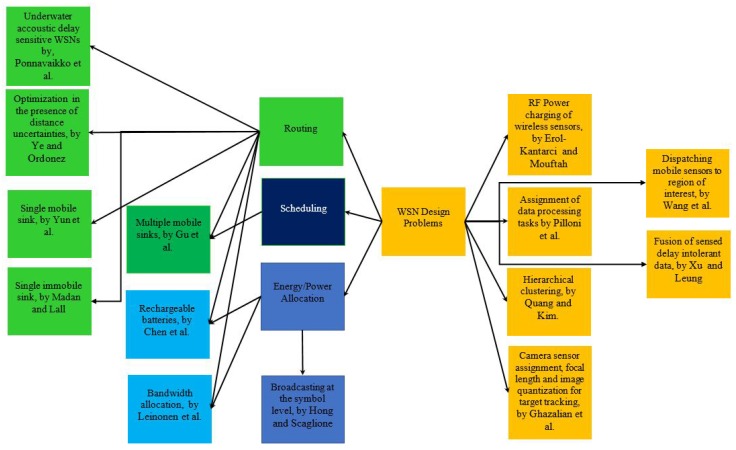
Taxonomy of the wireless sensor network (WSN) design problems considered in this paper.

As the taxonomy in Figure 1 shows, there are a number of different types of design problems that involve routing as a common key design aspect, possibly jointly with others like power allocation or scheduling in a subset of those. There are many papers in the WSN literature that have considered routing as a design aspect and is still being considered in recent research works like [16,17,18]. For this reason, in part II of this paper, we focus specifically on linear network problems and their algorithms, which deal with routing of network flows. Figure 1 shows that other than routing in different variants of WSNs, we also have selected topics that are necessarily not connected, like RF power charging, visual tracking etc. The main aim here is to illustrate and discuss how optimization is used in a wide number of applications, topologies (single sink, multiple sink, mobile sinks, etc.) and design aspects of WSNs. There are certainly many more in the literature that the readers can come across in the literature.

This paper extends our recently published conference paper [19], which was a shorter version and considered only the 7 routing problems of the above 14 problems. The main approach we take is to identify how each design problem is formulated into an optimization problem. Some of the problems were reformulated later by the authors to make solution methods more efficient. We discuss both the first formulations and the reformulations and provide comments about both cases for each problem. Later we discuss the advantages and disadvantages associated with each formulation and then discuss the solution methods. While we give clear scientific reasons and arguments to support our views about each formulation, most of the comments are definitely based on our opinions and strongly influenced by our understandings of the models as presented by the authors of the papers reviewed. Our goal is to present a tutorial on the subject.

Optimization techniques that have been in the operations research (OR) literature for almost a century provide a rich reservoir of different types and classes of optimization problems that have been studied extensively. For these, different solution techniques are available that have experienced development over the years until they have reached to a mature level in which their computational and storage performance have been extensively tested and assessed. Among these are the different variants of Lagrangian relaxation [20], dual decomposition methods [21] column generation [22] and many others. We dedicate part II to explain *linear optimization* since this was the first type of optimization that appeared and lots of special structured linear programs have been studied for which efficient algorithms have been developed. Moreover, linear optimization forms the basis of *non-linear optimization* and other advanced optimization types like *mixed integer problems* and a large number of WSN problems can be formulated as linear optimization problems.

In the linear optimization part, we explain classical special structured problems like *minimum spanning tree*, and *network flow* problems as these seem to us to be highly useful when it comes to WSNs. We state our own examples of how these classical linear special structured problems can be used to model, and hence solve, design problems in WSNs. Nonlinear programming also receives some good attention in this tutorial paper we focus on its theoretical concepts and present some of the different methods available in the OR literature. Finally, for part II of the paper, we discuss *decomposition* solution schemes, which are suitable for linear or non linear optimization problems. Decomposition schemes are attractive for WSNs, which are mainly of ad-hoc nature i.e., no central controller responsible for managing the resources for communications purposes, and have been used in some of the papers we consider in this tutorial. Being able to decompose the optimization problem into a number of smaller ones that can be solved separately by each sensor node, would be highly desirable to reduce the computational effort compared to centrally solving the problem. Since we target researchers who are new in using optimization techniques, we strongly believe that part II of this paper is necessary to understand some of the techniques used in part III of this paper.

Distributed algorithms are highly desirable since a sensor node has limited processing capabilities, and hence using one node to solve the problem would consume too much processing energy. Moreover after the problem is solved, the components of the solution specific to each node need to be transmitted to the respective nodes. For a centralized scheme, these transmissions will be from the solving node to all the nodes in the network, while in the distributed scheme these will only be to a subset of nodes (usually the neighbors only). Therefore the communication overhead in distributed algorithms are lower.

We believe that in order to exploit the full strength of the extensive tools in the optimization literature, good formulation is necessary to reduce the design problem to one of the classical optimization problems for which those well studied solution techniques could be used. In this paper, such techniques are illustrated for the different design problems in WSNs mentioned earlier, for which we have selected a paper for each. One section is dedicated to explain each design problem which is further divided into subsections that emphasize on the following:

- the system model and design objectives,
- problem formulation,
- any reformulation methods,
- solution methods,
- any important results,
- our comments on some or all of the above.

The purpose of our observations and comments on some of the problems is to shed some light on the points that we believe should be taken into account in the future when using optimization techniques for solving WSN design problems. As much as we could we used similar notation and definitions to those used in the corresponding papers to make it easy for the reader to relate to them if they wish to get back to them for more details.

## Part II Overview on Mathematical Optimization

This part of the paper serves as the fundamentals of optimization techniques, required for being able to use optimization techniques in any WSN design as part III shows. The purpose of this part (part II) is to give a basic tutorial on optimization techniques. Section 1 provides a brief overview on the history of operations research. Section 2 provides an introduction to optimization and the different classes of optimization problems. Section 3 is focuses on linear programming, which is the first type of optimization that was considered in the optimization literature and whose concepts form the foundations of more advanced optimization problems. The simplex algorithm, linear duality, Karmarkar’s algorithm, dual simplex as well as a discussion on computational efficiency of simplex and Karmarkar’s algorithm are all provided in Section 3.

Section 4 discusses a special class of linear programs that is very important for WSNs. This type of problems is known as the *network flow problems* and the *minimum spanning tree* MST problem. Section 4 also presents examples on how these types of problems and their extremely efficient algorithms can be used in WSN design problems. Section 5 provides an introduction to non-linear programming (NLP), which is a very vast field of optimization problems. We discuss fundamental concepts like Lagrange functions and multipliers, KKT conditions, convexity, duality in NLPs etc. These form the basis of any algorithm design designed to solve an NLP. A taxonomy of the different solution methods used in NLP is presented in a summarizing figure. The details of each are beyond the scope of this paper though.

Finally, Section 6 presents primal and dual decomposition schemes for solving optimization problems, which enable us to break problems that are, partially decomposable except for a subset of complicating constraints or variables. Hence, a large problem can be broken down into a number of smaller subproblems and a master problem that can be solved separately in iterative fashion in a distributed scheme. Distributing the computations for the optimization problem across a large number of sensors yields alleviates the computational effort (and corresponding battery energy) that a single sensor node will expend in a centralized scheme.

## 1. The History of Optimization

The beginning of the science of optimization techniques, also known by the names *Operations Research* (OR) and *Mathematical Programming*, dates back to the early years of the World War II. At that time, it was extremely necessary to allocate scarce resources to the different military operations and to the activities for each operation in an efficient manner. For that purpose, the British and then the U.S. military management asked a large number of scientists to apply a scientific approaches for that purpose. In other words, they were asked to do *research* on military *operations* [23]. These teams of scientists were the first OR teams. By developing effective methods of using the the newly invented radar at that time, these teams had an important role in winning the Air Battle of Britain. Through their research on how to better manage convoy and antisubmarine operations. Similar efforts played a major contribution in winning the Battle of the North Atlantic.

When the war ended, the success of OR in the military field boosted interest in applying it other fields too. As industries started to boom after the war, the problems caused by the increasing complexity and specialization in organizations were arising. These seemed basically the same problems as the ones that used to be considered by the military but in the context of a different field. By the early 1950s, researchers had introduced the use of OR to a variety of organizations in business, industry, and government. The rapid spread of OR soon followed.

There were two key aspects that led to the development of optimization methods [23]:

- A large progress was made early in improving the techniques of OR. After the war, many of the researchers who had participated on OR teams or who had heard about this work were motivated to continue research in that direction, which lead to advancements in the state of the art. A leading example is the simplex method for solving linear programming problems, developed by George Dantzig in 1947.
- Computer revolution which lead to the development of electronic computers. This is because a large amount of computations is usually required to deal most effectively with the complex problems typically considered by OR. Doing this by hand would be impossible, besides the fact that in most of the practical problems, deriving a closed form expression for the solution is not possible neither.

## 2. Introduction to Mathematical Programming

In a mathematical programming problem, the decision maker wishes to choose decision variables to maximize or minimize an objective function, subject to the requirement that the decision variables satisfy certain constraints. In the case of WSNs, the decision maker can be one or more sensors, for e.g., cluster head sensor nodes. The objective function is basically a design criterion that we are trying to achieve, e.g., maximum throughput, minimum delay, maximum energy efficiency etc. The constraints can be physical restrictions like amount of available battery power, a subchannel capacity or a maximum allowable transmit duration etc.

There are four broad categories of optimization problems, where for each category, an extensive literature of different methods to solve certain sub categories have been proposed. These categories are:

- *Linear Programming* (LP): This is the first category of optimization problems that were considered by early scientists and researchers in the OR field. Basically the decision variables are continuous, the objective function and all the constraints are linear in the decision variables. We would recommend the references [23,24,25] as an introduction with many solved examples for LPs.
- *Non-Linear Programming* (NLP): In this category, we only have continuous variables but either the objective function or at least one of the constraints are non linear in the decision variables. Good references are the text book by the convex optimization pioneers S. Boyd and V. Vandenberghe [26] and Boyd’s notes for his Convex Optimization I (EE364a) and Convex Optimization II (EE364b) courses in the Stanford University [27,28].
- *Mixed Integer Linear Programs* (MILP): This is a linear program, where a subset of the decision variables have the restriction that they can only take a set of integer values for each. Again the three textbooks in [23,24,25] would be a good reference for an introduction to this type of optimization problems.
- *Mixed Integer Non-Linear Programs* (MINLP): This is a non linear program in which a subset of the decision variables must take integer values only. A good reference for this type of problems is the monograph in [29], which is a compilation of key MINLP papers published in a number of strong journals in the field of Operations Research. Another can be a textbook by the global optimization pioneer C.A. Floudas [30].

In all of the above broad classes of optimization problems, decision variables should completely describe the decisions to be made. In the case of WSNs in particular, the following can be an example of what the decision variables can be used to model:

- The amount of radio power at a particular time slot a sensor is going should transmit (power management), this is usually modelled using continuous variables.
- The time instants and/or durations for each sensor node’s transmission (scheduling). Both continuous and integer variables have been used for that purpose.
- The amount of spectrum bandwidth to be used by each sensor node for transmission. Both continuous and integer variables have been used for that purpose.
- The set of links to be used in a WSN. Binary variables have been used to model whether a link would be used or not.
- Modulation schemes, and channel coding rate each node can use on any of its links. Integer variables are mostly a suitable choice for modeling that aspect.
- The data flow rate in bps per link, usually modelled using continuous variables.

## 3. Linear Programming Problems

We strongly believe that it is important to understand linear programming very well, if a researcher intends to use optimization as his research tool in WSNs or in any design problem. Concepts in linear programming like linear duality, sensitivity analysis, complementary slackness and shadow prices are needed to understand NLPs and other advanced optimization techniques. Therefore, to have a solid understanding of NLP, a researcher who would like to lay their own foundation in using the tool is strongly advised not to bypass LP.

Linear Programs (LPs) were the first type of optimization problems to be considered, and as mentioned earlier, they date back to the days of World War II. It is for that class of problems, George Dantzig proposed the *simplex* method to find the optimum solution efficiently. Basically any LP can be represented in the form:

(1a) $$z=\underset{x}{min}\;c^{T}x$$

s.t.

(1b) Ax≤b,

(1c) $$x_{LB}\leq x\leq x_{UB}$$

where $x\in R^{n}$ is the vector of *n* decision variables, $c\in R^{n}$ is the vector of costs per unit of the corresponding decision variables, $A\in R^{m\times n}$ is the coefficient matrix of all the constraints, $b\in R^{m}$ represents the vector of values a constraint must not exceed, $x_{LB}$ and $x_{UB}$ are vectors that hold the lower and upper bounds on each decision variable in x.

Generally speaking, the constraint set given by Equation (1b) either:

1. geometrically forms a polyhedron *P* of a set of infinitely many feasible solutions in the space of the decision variables i.e., $P\subset R^{n}$, or
2. no solution at all for which we say the LP is *infeasible*.

An optimal solution for a feasible bounded LP is known to occur at a vertex point of the polyhedron representing the feasible region. We can have infinitely many optimal solutions when, for example, two vertices yield optimal solutions, then all points falling on the edge connecting them would return the same value of the objective function, hence all are optimal. The reader would probably guess that a key idea for devising an algorithm that solves general LPs, is to move around the vertices of the polyhedron of feasible solutions. In fact, that’s a correct guess, as the classic simplex algorithm relies in its core on this idea.

### 3.1. Solving LPs

There are two main methods for solving generic LPs (i.e., LPs whose coefficient matrix has low sparsity). The first one that was invented in 1947 was the simplex algorithm and its variants that came later like the dual simplex and revised simplex. The second is the *Karmarkar’s algorithm* which falls under the category of interior point methods [31] which appeared in the 1980s. Both are being used by today’s solvers like the optimization package developed by IBM, CPLEX.

#### 3.1.1. Simplex Method

The simplex algorithm first needs a reformulation to be done to the LP in Equation (1), to put it in the *standard form*, before it can be applied. In optimization solvers, this is done automatically at a preprocessing stage, provided you provide the problem to the solver in the form in Equation (1). Basically the standard form of any LP requires representing all inequality constraints in the form of inequality constraints by the introduction of nonnegative *slack* and *excess* variables. Any unrestricted variable $x_{i}$ gets replaced by two variables such that $x_{i}=x_{i}^{\prime }-x_{i}^{\prime \prime }$ and $x_{i}^{\prime }\geq 0,x_{i}^{\prime \prime }\geq 0$. A simplex *tableau* is first constructed [24], for an optimization problem in Equation (2), the initial tableau is given by Table 1.

(2a) $$max\;\;z=60x_{1}+30x_{2}+20x_{3}$$

s.t.

(2b) $$x_{1}+6x_{2}+\;x_{3}\leq 48$$

(2c) $$4x_{1}+2x_{2}+1.5x_{3}\leq 20$$

(2d) $$2x_{1}+1.5x_{2}+0.5x_{3}\leq 8$$

(2e) $$x_{2}\leq 5;x_{1},x_{2},x_{3}\geq 0$$

Then the simplex algorithm needs a *basic feasible solution* (**bfs**) to start from, in which all the variables in the standard form LP are non-negative. In case the LP has only (≤) constraints, slack variables become the set of *basic variables* and are assigned initial values equal to the right hand side values of the corresponding constraints as in Table 1. The rest of the decision variables are initially in the set of *non-basic variables*, i.e., take the values of zero. If there are any (≥) constraints, then two of the methods that can be used to find a starting **bfs**, are the *Big-M* method and the *Two Phase Simplex* method [23,24,25].

The simplex algorithm utilizes the Gauss-Jordan algorithm which originally solves a system of linear equations. Since we have many solutions for the system of linear equations for an LP formulation in the standard form, the simplex uses the Gauss-Jordan algorithm to move across **bfs**s that are adjacent to each other as long as the objective function value (*z* in the tableau) decreases. In each iteration, elementary row operations (eros) are performed to obtain the improving **bfs**. If in a given iteration, the coefficients of all non-basic variables in the objective function row in the tableau (row 0 usually) are negative (assuming we are considering a minimization problem), then current **bfs** is optimal. If any variables in row 0 have negative coefficients, the simplex algorithm chooses the variable with the most negative coefficient in row 0 to enter the basis (this variable is called the entering variable) and (eros) make it a basic variable. The variable that leaves the basis to become a non-basic variable and the new value of the entering variable are decided by a ratio test [24].

#### 3.1.2. Karmarkar’s Algorithm

This method requires putting the LP to be solved in the following form:

(3a) $$\underset{x}{min}\;c^{T}x$$

s.t

(3b) Kx=0

(3c) $$1^{T}x=1$$

(3d) x≥0

The point $x^{0}=\frac{1}{n}1$ is required to be feasible and the value of the objective function at an optimal solution is required to be zero. Karmarkar’s method uses transformation from projective geometry to create the transformed variable vector **y**. The transformation given by a function $f\left(x\right)=y$ always projects the current point into the center of the feasible region defined by **y**. The algorithm starts in the transformed space by moving from $f\left(x^{0}\right)$ in a direction that decreases the objective function value while maintaining the feasibility, obtaining the point $y^{1}$ which is close to the boundary of the feasible region. The new point satisfies $f\left(x^{1}\right)=y^{1}$. This keeps repeating until the objective function value of Equation (3) for $x^{k}$ is close enough to 0. In the transformed space, the algorithm is always moving away from the center of the feasible region.

#### 3.1.3. On the Efficiency of Simplex and Karmarkar’s Algorithms

Efficiency is measured in terms of the computational time required by a CPU to obtain an optimal solution. Theoretically, one of the measures for that is the worst case complexity, usually denoted by the big ’O’, and is a function that is proportional to the time of the number of required operations (or iterations) versus the size of the problem. The size of the problem is described by the number of decision variables *n* and the number of constraints *m*.

Theoretically, the worst case number of iterations required by the simplex algorithm is bounded by the number of possible **bfs** which is nm. This could be really big for a large problem. Fortunately, practical experience with simplex indicates that the optimal solution can be found in less than 3m **bfs**s. Karmarkar’s algorithm, on the other hand, is a polynomial time algorithm which means that for an LP of size *n*, there exist positive numbers *a* and *b* such that it can be solved in a time at most $an^{b}$.

We need to always keep in mind that the theoretical complexity is an upper bound function (since it is for the worst case) of the solution time required versus the problem size, which can be a loose bound. Therefore for an exponential time algorithm this may not be quite a reliable prediction of how they will perform in practice. For the theoretical time complexity of an algorithm to be reliable, especially if it’s worst case is exponential, we will need rigorous analysis of the algorithm as a stochastic process to derive at least a statistical average and variance of the algorithm. However this is something we have not come across in the optimization literature before, which may indicate that it is not commonly done. Therefore, in our opinion, extensive numerical experiments are needed to evaluate the performance of an algorithm whose theoretical worst case complexity is exponential, in order to evaluate its computational efficiency.

### 3.2. Linear Duality

Associated with any LP is another LP called its *dual*. Knowing how both are related is very important for understanding advanced linear programming and non-linear programming. It gives insights in another important topic which is *sensitivity analysis*, which studies the effect of changing some of the costs in the objective function, or the bound of a constraint function on the feasibility and optimality of an obtained optimal solution. Sensitivity analysis is extremely important when the parameters of an LP have non-zero uncertainty. In the field of WSNs, this can happen in the measurements of the channel coefficients, which have could have some degree of error.

There is a dual variable (or *dual price*) associated with each constraint in the primal problem. It represents the objective function improvement obtained by relaxing a binding constraint by one unit. A dual variable is nonzero only when the constraint is binding and zero otherwise. This is known as the *complementary slackness* property connecting the primal and dual problems. The units of the dual prices are the units of the objective function divided by the units of the constraint. Knowing the units of the dual prices can be useful when you are trying to interpret what the dual prices mean.

The dual of the *primal LP* given in Equation (1) (with a the minor modification of having x≥0) is:

(4a) $$w=\underset{y}{max}\;b^{T}y$$

(4b) $$A^{T}y\geq c$$

(4c) y≥0

where $y\in R^{m}$, and the set of variables $y_{i}$ are the dual variables.

In linear duality, the *dual theorem* states that the optimum objective function value of both the primal and its dual problem are equal, if the primal has a feasible bounded solution. This is an interesting property in its own, as well as a number of important insights the dual theorem has in linear programming.

Important elements that constitute the dual theorem are:

1. *Weak Duality*: For any feasible solution x to the primal problem given in Equation (1) and any feasible solution y for Equation (4), the (z-value for x) ≥ (w-value for y). If a feasible solution to either the primal or the dual problems is obtained, weak duality enables its use to obtain a bound on the objective function value of the other problem.
2. *Strong Duality*: Is a situation when the bounds are equal to each other i.e., $c^{T}x^{*}=b^{T}y^{*}$, where $x^{*}$ and $y^{*}$ are optimum solutions of the primal and dual respectively.
3. If the primal is unbounded the dual is infeasible, and vice versa.
4. A basis BV in a primal problem that is feasible is also optimal **if and only if** the vector $\bar{y}=c_{BV}B^{-1}$ is dual feasible, where:
  (a) $c_{BV}$ is a 1×m vector of costs in the objective function of the standard form of the primal problem (1) that corresponds to the basic variables in the optimal tableau (last tableau obtained) and,
  (b) B is an m×m matrix whose *j*th column is the column for the *j*th basic variable in the standard form of the primal problem (1).
  Hence, when the optimal solution $x^{*}$ for the primal problem is found by the simplex, we also have found the optimal solution $y^{*}$ to its dual problem.

#### 3.2.1. The Dual Simplex

When the simplex method solves a primal maximum problem, we begin with a primal feasible solution but a dual infeasible solution. Through the sequence of simplex iterations, the primal feasibility is maintained all the time. An optimal primal solution is only obtained when it is also dual feasible (yields useful insights in sensitivity analysis [24]). In many situations it could be more efficient to solve an LP by beginning with a tableau (of a maximization problem) in which each variable in row 0 has a non-negative coefficient and at least one constraint has a negative right-hand side (RHS), i.e., primal infeasible.The dual simplex maintains the non-negative reduced costs (dual feasibility), and when it obtains a tableau with non-negative RHS (primal feasibility), an optimal solution is achieved.

The main steps of the dual simplex for a maximization problem are as follows:

1. Check the sign of the RHS of each constraint. If all are negative then an optimal solution is at hand. Otherwise, at least one constraint has a negative RHS and we go to step 2.
2. The most negative basic variable leaves the basis. Select the variable to enter from the row in which the leaving variable is basic (pivot row) using an absolute ratio test. The variable with the maximum absolute value of the corresponding coefficient in row 0 divided by its coefficient in the pivot row.
3. An infeasibility is indicated if there exists a constraint for which the corresponding RHS is negative and the coefficient of each of its variables is negative in the current tableau. If the problem is feasible we go back to step 1.

## 4. Network Flow Programming Models

This section serves as a foundation for many types of optimization problems in WSNs, specifically, routing problems. Many of the routing design problems in WSNs problems are formulated as network flow problems. The examples that we provide later in part III of this paper, also include non-linear network flow problems. In order to understand the basics of this class of optimization problems, We believe it is very important to provide the linear network flow problems and their special cases, since nonlinear network flow problems extend the concepts in linear ones.

A network is a set of nodes joined by a set of arcs which carry flow, where the total flow entering each node must equal the total flow leaving each node. One optimization problem that is defined on a network is to determine the assignment of flows to the arcs such that the total cost of the flow is minimized. This is called the network flow programming (NFP) problem or, alternatively, the minimum-cost flow problem. The cost in WSNs could be the average end to end delay from a sensing node to a sink node when considering a delay sensitive design problem. Another possibility can be the amount of battery energy consumed in all sensor nodes while performing RF transmissions for routing data to a sink node. Also, the network flow problem can be a maximization problem in which we desire the maximization of the throughput for all sink nodes.

**Figure 2:**
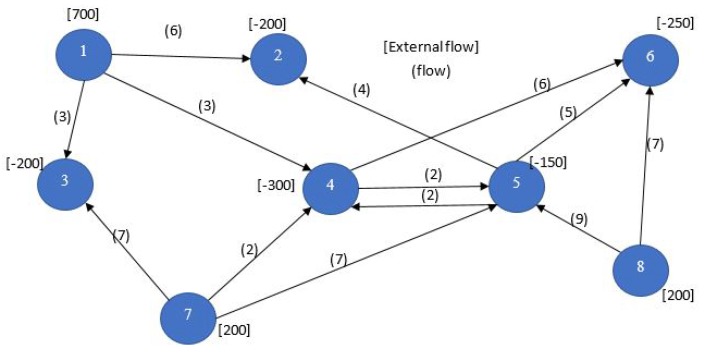
Example of a network flow programming model.

**Figure 3:**
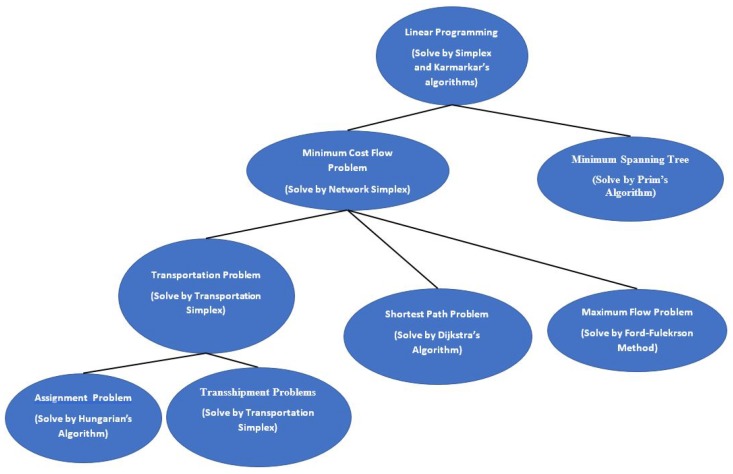
Taxonomy of the relationships of Linear Programs (LPs), network flow programs (NFPs) and their special classical sub-classes [25].

NFP can be considered a special case of LP, where an equivalent graphical model is more appropriate and suitable in modeling the LP network problem due to its special structure. Problems that can be modeled with NFP include some of the classical problems of optimization such as the assignment problem, the shortest path problem, the maximum flow problem, the pure minimum-cost flow problem, and the generalized minimum-cost flow problem. The network is a compact graphical representation that allows the analyst to quickly visualize the essence of the problem as shown in Figure 2 for example. When a situation can be modeled entirely with NFP, very efficient algorithms are available for solving the associated optimization problem, many times more efficient than the standard simplex method with respect to computational effort and storage requirements. Figure 3 shows a taxonomy of the different NFP subclasses as well as a popular special structure problem known as the *minimum spanning tree problem*. The figure shows their inter-relations and the corresponding solution algorithms. Further explanation is provided on those later in this section.

### 4.1. Terminology

The following are the main the terms used when referring to linear network problems [25]:

1. **Nodes and Arcs**: The network flow model consists of nodes and arcs. In the context of modeling a problem, each node, shown as a circle, represents some aspect of the problem, such as a physical location, an individual worker, or a point in time. In WSNs it is commonly the sensor, or could refer to the sensor node in a number of discrete spacial positions if the sensor is mobile for example [3]. Arcs are directed line segments that generally pass from an origin node to a terminal node, although in the case of external flow, an arc may be incident to only one node. If an arc does not have a direction, it is sometimes referred to as an edge.
2. **Arc Flow**: Flow is associated with the network, entering and leaving at the nodes and passing through the arcs. The flow in arc *k* is $x_{k}$. When flow is conserved at the nodes, the total flow entering a node must equal the total flow leaving the node. The arc flows are the decision variables for the network flow programming model.
3. **External Flows**: The external flow at node *i*, denoted by $b_{i}$, is the flow that must enter node *i* from sources, or leave node *i* for destinations outside the network. A positive external flow enters the node, and a negative external flow leaves the node. In the network diagram in Figure 2, the external flow is shown in square brackets adjacent to the node. External flows can model for e.g., the required data rate a sensor node should receive when it is the destination of a particular transmission. Also, they can model the source rate of a wireless sensor node that senses an external phenomena and converts it to electrical signal, then encodes it to a digital bit stream having specific bit rate.
4. **Upper and Lower Bound on Flow**: Flow is limited in an arc by lower and upper bounds. Sometimes the term capacity refers to the upper bound on flow. In wireless sensor networks, the capacity depends on the received signal to noise ratio (SNR) at the node on which the flow arc converges to, given the assumption that the network uses *time division multiple access* (TDMA), and hence a noise limited system. If radio power control is considered in the problem, the capacity becomes a nonlinear logarithmic function in the power decision variable. This is an example of a possible non-linear network *flow and capacity* problem in a WSN. The capacity of an arc *n* is given by $c_{n}=\Delta B_{n}log_{2}\left(1+g_{n}p_{i}\right)$, where $\Delta B_{n}$ is the bandwidth for the link represented by arc *n*, $g_{n}$ is the channel gain for the link and $p_{i}$ is the transmitted power on the link, given that $n\equiv \left(i,j\right)$. In the case power management is not considered, then the transmission power becomes constant instead of a decision variable, and the entire link capacity becomes constant.
5. **Cost**: The criterion for optimality is cost. Associated with each arc *k* is the cost per unit of flow $c_{k}$. Negative values of $c_{k}$ correspond to revenues. In WSNs, this can be equivalent to the battery energy expended per flow, where our flow would be in bits per second (bps). Therefore the cost has the units Joules-per-bit. In this case, we are assuming the power to be constant, i.e., we have constant arc capacities. A modulation scheme which decides on the number of bits per second is our decision variable on the flows.
6. **Gain**: The arc gain $G_{k}$ multiplies the flow at the beginning of the arc to obtain the flow at the end of the arc. When a flow $x_{k}$ is assigned to an arc, this flow leaves the origin node of the arc. The flow entering the terminal node is $G_{k}x_{k}$. The arc’s lower bound, upper bound, and cost all refer to the flow at the beginning of the arc. Gains less than 1 model losses while gains greater than 1 model growth in flow. In the case of WSNs, the arc gain can represent the reciprocal of the path loss, and the flow could be the radio power transmitted by every sensor in the network for a power management problem.

A network in which all arcs have unit gains is called a pure network. The optimal solution for a pure network with integer parameters always has integer flows. If some gains have values other than 1, the network is a generalized network, and the solution is not usually integral.

### 4.2. Special Classes of NFPs

It is very useful to recognize several special cases such as the models for the transportation, shortest path, and maximum flow problems. These models differ primarily in the set of parameters that are relevant, or in the way the nodes and arcs are arranged in the flow diagram.

#### 4.2.1. Transportation Problem

**Figure 4:**
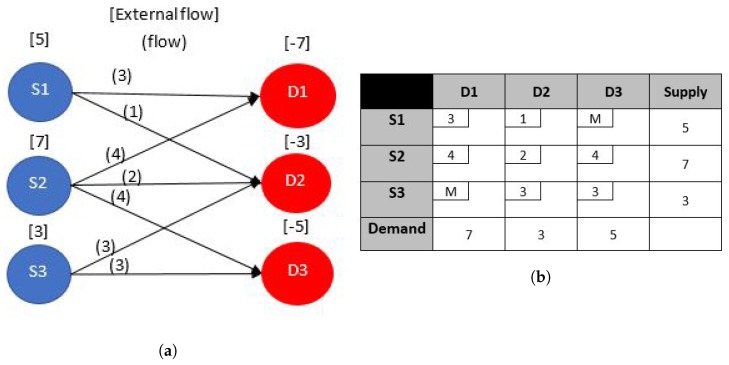
An illustrative example of a simple transportation problem. (**a**) NFP model of the transportation problem; (**b**) Tableau of a transportation problem.

This type of network has a *bipartite structure*, that is the nodes are grouped in two sets and the arcs can only be between nodes in different sets. To understand this class of problems we explain it by an example from [25]. Consider the network in Figure 4a. This problem deals with a set of sources, labeled S1, S2, and S3 at which supplies of some commodity are available, and a set of destinations, labeled D1, D2, and D3 where the commodity is demanded. Shipping costs are specified in the transportation tableau in Figure 4b, and the supply and demand data are shown outside the tableau. Supply and demand data are shown along the margins of the tableau and are denoted symbolically as $s_{i}$ and $d_{j}$, respectively, where i=1,…,m and j=1,…,n. In a classical transportation problem, the problem is *balanced*, which means that the total supply always equals the total demand—i.e $\sum_{i}^{m}s_{i}=\sum_{j}^{n}d_{j}$. This is the case in Figure 4, and is a sufficient condition for feasibility. Unbalanced instances of the problem can always be put into this form with the addition of a dummy supply point (call it m + 1) when demand exceeds supply, or a dummy demand point (call it n+1) when supply exceeds demand. If Δ denotes the excess, we set $s_{m+1}=\Delta$ or $d_{n+1}=\Delta$ depending on the imbalance.

The **transportation simplex algorithm** is a variant of the simplex algorithm that exploits the structure of the transportation tableau to reduce the computational effort as compared to the generic simplex. Actually, applying the simplex algorithm to a transportation problem would be inefficient as we could encounter *degenerate* **bfs** [25]. In this case, there is a chance of *cycling* to occur, which is a situation in which the the algorithm moves from one **bfs** to other adjacent ones that yield the same objective function value and then comes back to the one it started from. i.e., A−B−D−C−A−B−D−C−A….. The algorithm then gets stuck there until a time limit or iteration limit is hit.

In transportation simplex, an initial **bfs** can be obtained by the *Vogel’s method*, the *Northwest corner method* or *Russell’s method* [24], both are much efficient for a transportation problem when compared to the Big M or the dual phase simplex methods. If the researcher is able to recognize that a particular design problem in WSNs can be formulated into a transportation problem, they can benefit from the more efficient transportation simplex instead of using the normal simplex. Good and clear explanation on how the transportation simplex operates and how it exploits the transportation tableau’s special structure can be found in [24,25]. The implementation of of the transportation simplex is easy, as it involves only addition and subtraction operations, a C++ implementation code for the algorithm can be found in [32]. **Example for a Possible Application to WSN**: This class of problems can be used to formulate a simple WSN network where there are three source sensors S1, S2, and S3, that sense different things, like temperature, humidity and pressure. There are possibly also the sink nodes D1, D2 that connect the WSN to another network, the Internet possibly. The arcs would represent the possible direct transmissions each source sensor node can make to any of the three sinks. The presence of a possible arc could depend on the radio transmission power level, meaning that the sink at which a particular arc is incident on is in the transmission range of the source node. The costs of the arcs can represent the battery energy that would be expended per bit of flow on each arc. This is directly related to the distance between the sensors and the sinks. The objective would be to reduce the sum of all the expended energies per second overall arcs while meeting the rate demands which the sink nodes should be receiving.

#### 4.2.2. Assignment Problem

**Figure 5:**
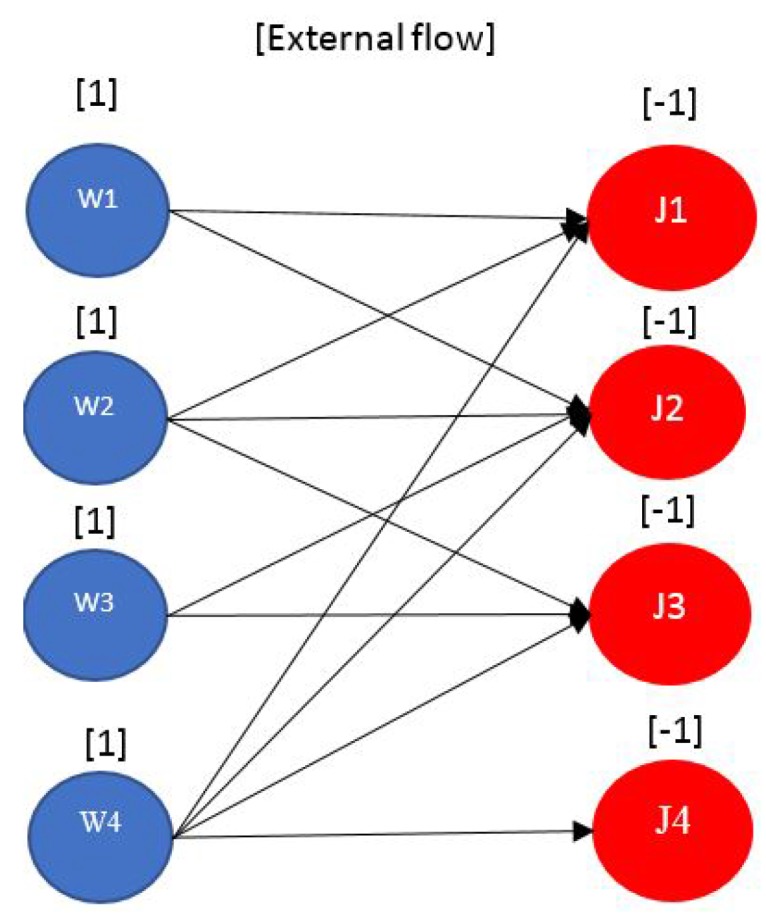
NFP model of the assignment problem.

This problem is very similar to the transportation problem except that the external flows are all +1 and –1. The only relevant parameter for the assignment model is the arc cost. Note that this model also has the bipartite structure, and an illustrative example is provided in Figure 5 which describes the network graph of a problem were a number of workers are required to be assigned to a number of jobs. The number of workers and jobs are equal, and the assignment is one-to-one. The decision variable for the assignment problem is denoted by $x_{ij}$, which takes the value 1 if worker *i* is assigned to job *j* and 0 otherwise. The each arc has a cost, which represents the cost $c_{ij}$ required for assigning *i* to job *j*. The objective is to find the assignment that yields the minimum cost $\sum_{i=1}^{m}\sum_{j=1}^{n}c_{ij}x_{ij}$. In case a problem is unbalanced (i.e., n≠m), it should be balanced by adding dummy nodes as explained in [24,25]. The problem has a transportation tableau for which the entries are either 0 or 1. Although the assignment problem is a special case of the transportation problem, but the transportation simplex turns to be inefficient for the assignment problem. A well known algorithm that has a cubic polynomial worst cast time complexity, $O\left(n^{3}\right)$, for the one-to-one balanced assignment problem is the *Hungarian Algorithm*.

**Example for a Possible Application to WSN**: One example we can think of, in which the assignment problem could be relavant in a WSN, is the allocation of subchannels to pre-assigned links in the network, assuming we have frequency selective fading. The subchannels, thus have different gains for different links in the network at different carrier frequencies. A frequency selective fading environment could be a place that have so many reflectors, like inside factories with many metal surfaces (a typical place were WSNs can exist). In such a place the delay spread would be high yielding a frequency selective fading channel. An optimization problem would be needed to *assign* the carrier frequencies (or subchannels) to the links. The cost for allocating a subchannel to a link can be the amount of attenuation experienced for the pair. The objective would be to minimize the overall attenuation in the network. Over here, we assume that we only assign one subchannel for each link, which means that the radio transmitter is a single carrier transmitter.

#### 4.2.3. Transshipment Problem

A transportation problem allows only shipments that go directly from a supply point to a demand point. In many situations, shipments may be allowed between supply points or demand points. Sometimes there may also be points, called transshipment points, through which goods can be transshipped on their journey from a supply node to a demand node. Basically a transshipment node differs from a supply or demand node in that it can both receive and send goods. This type of shipping problems is known as transshipment problems, for which the optimal solution can be obtained by solving a transportation problem.

**Figure 6:**
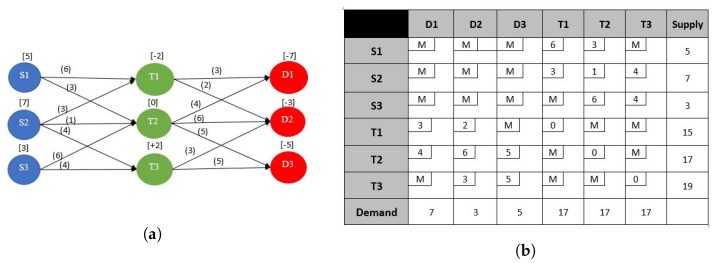
An illustrative example of a simple transportation problem. (**a**) NFP model of a transshipment problem; (**b**) Tableau of a transshipment problem.

In general we say that there are *m* pure supply points, *n* pure demand points, and *p* transshipment points. For an NFP model, a pure supply point would have only leaving arcs whereas a pure demand point would have only entering arcs. We let $s_{T}$ be the total supply available for the problem i.e., $s_{T}=\sum_{i=1}^{m+p}s_{i}$, and we let $t_{k}$ be the net stock position at transshipment node *k* for k=1,…,p. If stock is supplied at node *k*, then $t_{k}$ is positive, and if stock is demanded at node *k*, then $t_{k}$ is negative. In the example in Figure 6, m=n=p=3, with $s_{T}=17$. The basic steps for solving a transshipment problem would be:

Figure 6a illustrates the graph of an example of a transshipment problem. It can be noticed that the transshipment problem has an NFP model that is not bipartite. However, a transshipment problem can be transformed into a balanced transportation problem yielding an associated NFP model that is bipartite, whose tableau is illustrated in Figure 6b.

1. If necessary, put the problem into balanced form by adding either a dummy supply point to meet the excess demand or a dummy demand point to absorb the excess supply. Shipments to or from the dummy node will have zero shipping cost.
2. Construct a transportation tableau with m+p rows, one for each supply point and transshipment point, and n+p columns, one for each demand point and transshipment point. Each pure supply point *i* will have a supply equal to its original value $s_{i}$. and each pure demand point *j* will have a demand equal to its original value $d_{i}$. Each transshipment point will have a supply equal to $s_{k}=t_{k}+s_{T}$ and a demand equal to $d_{k}=s_{T}$.
3. A large cost *M* is assigned to shipments that are not permissible. A shipment is allowed from a transshipment point to itself and assigned a unit transportation cost of zero i.e., include $x_{kk}$ in the model and set $c_{kk}$ equal to zero for k=1,…,p. The transformed model for the example is shown in Figure 6b.
4. Obviously, the transportation simplex can then be used to solve the problem.

**Example for a Possible Application to WSN**: This kind of problem can be used to model a WSN sensor network where the source nodes are the ones that do the sensing of the required phenomena and the demand points are the sink nodes, as earlier explained for the classic transportation problem. The transshipment nodes can be relaying sensors that receive and forward the transmission from one sensor node to another as in Figure 6. The objective can be the minimization the of overall delay where the cost $c_{ij}$ of of each arc represents the amount of delay per unit throughput, i.e., ($s^{2}/b$), and the decision variable $x_{ij}$ represents the flow in bps, The demand constraints represent the target throughput each sink node should receive. The objective function is the sum of the delays $c_{ij}x_{ij}$ on all arcs.

#### 4.2.4. Shortest Path Problems

The length of a path in a network is the sum of the arc costs along the path from a source node to a destination node. The problem is to find the shortest path from some specified node, to some other node or perhaps to all other nodes. The latter problem is called the *shortest path tree* problem because the collection of all shortest paths from a specified node forms a graph structure called a *tree*. Since it is not much more difficult to find the paths to all nodes than it is to find the path to one node, the shortest path tree problem is usually solved. The NFP equivalent of the shortest path tree problem is formed by equating arc distance to arc cost, assigning a fixed external flow of m−1 (*m* is the number of nodes) to the source node, and assigning fixed external flows of −1 to every other node. The *Dijkstra’s algorithm* is a classic algorithm that can be used to solve this problem to optimality in a worst cast time complexity $O\left(n^{2}\right)$, where *n* is the number of nodes. There are many references to understand how the algorithm operates, amongst those is [24].

**Example for a Possible Application to WSN**: In WSNs, a shortest path problem can be used to solve a routing problem were the arc costs could be the energy expended on a link and an objective of minimizing the overall energy in an entire path from a source node to each and every node in the network. Assuming constant pre-set transmission power for each sensor node, the energy that has to be expended over a link can be approximated (in a simple scenario) to be linearly proportional to the attenuation on the link. The problem would then be a routing problem, in which the set of arcs that comprise a complete path from the source node to each node has to be found. Each non-source sensor node would be receiving a separate stream of packets that are unique to each node (unicasting). The path between each pair of nodes that minimizes the transmission energy would be obtained by the Dijkstra’s algorithm.

#### 4.2.5. Maximum Flow Problems

Many situations can be modeled by a network in which the arcs may be thought of as having a capacity that limits the quantity of a product that may be shipped through the arc. In these situations, it is often desired to transport the maximum amount of flow from a starting point, which is the source node, to a terminal node which is the sink node. These problems are defined as maximum flow problems.

**Figure 7:**
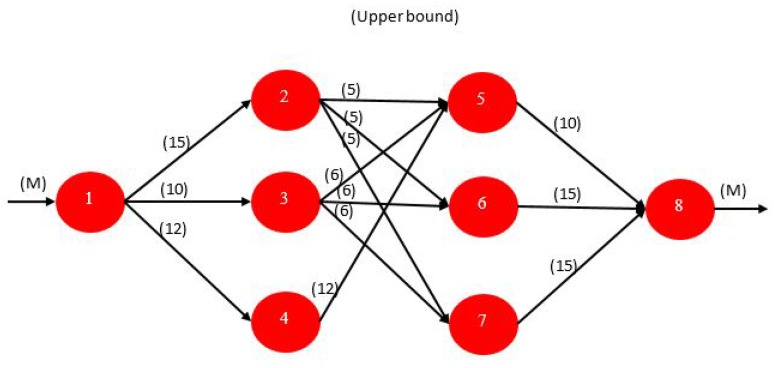
NFP model of a maximum flow problem.

The maximum flow problem again fits the NFP structure. With one exception, only the arc capacities or upper bounds are the relevant parameters. The problem is to find the maximum possible flow from a given source node *s* to a given sink node *t*. An NFP model with node 1 as the source and node 8 as the sink is depicted in Figure 7. All arc costs are zero with the exception that the cost on the arc leaving the sink is set to −1. Since the objective is to minimize cost, the maximum possible flow is delivered to the sink node. By convention, we assign a large number M to the upper bound on flow into the source and out of the sink node.

**Figure 8:**
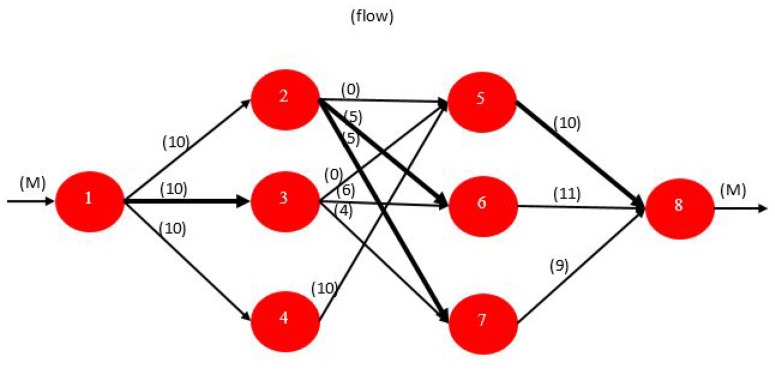
Maximum flow (minimal cut) = 30.

The solution to the example is given in Figure 8. The maximum flow from node 1 to node 8 is 30. The individual arc flows that yield this result are shown in parentheses. The bold arcs in the figure, (( 1,3), (2,6), (2,7), (5,8)), are called the *minimal cut*. In general, a cut is a set of arcs such that if they are removed from the graph, no flow can pass from the source to the sink. A cut partitions the nodes in a graph into two disjoint subsets or subgraphs. The arcs in the minimal cut are the bottlenecks that are restricting the maximum flow. The fact that the sum of the capacities of the arcs on the minimal cut equals the maximum flow in this example is not a coincidence. It is always the case and is a famous primal-dual result in network theory called the max-flow min-cut theorem. The arcs comprising the minimal cut can be identified using sensitivity analysis. This problem can be solved efficiently by a classical algorithm known as the *Ford-Fulekrson Method* which has a worst case complexity $O\left(n.m\right)$ where *n* is the number of nodes and *m* is the number of arcs.

**Example for a Possible Application to WSN**: An obvious possible application for this type of problems in WSNs is in routing traffic from a single source sensor node that senses a required phenomenon and transmitting the corresponding packets through the WSN where *bifurification* of packets are allowed, down to a single sink sensor node. The maximization of the flow here, would be a maximization of the throughput received by the sink from the WSN from the original source sensor node given the constant finite link capacities that are limited by the transmission power of each sensor node in the network, the channel gain and the channel bandwidths ( obtained from Shannon-Hartley relation). There are no relevant costs for the links over here, and each link is assigned one channel to transmit on, and is not reused by any other links.

#### 4.2.6. Minimum Spanning Tree (MST)

This type of problems aims to find the set of arcs in a graph network that connect all nodes such that the sum of the arc lengths is minimized. Such a group should contain no loop, obviously. For a network of *n* nodes, a *spanning tree* would be a group of n−1 arcs that connect all nodes of the network and contain no loops. A spanning tree of minimum length is a *minimum spanning tree*.

*Prim’s* algorithm is a greedy algorithm that finds a minimum spanning tree for a weighted undirected graph. Informally speaking, the way it works is as follows:

1. Initialize a tree with a single vertex, chosen arbitrarily from the graph.
2. Grow the tree by one edge: of the edges that connect the tree to vertices not yet in the tree, find the minimum-weight edge, and transfer it to the tree.
3. Repeat step 2 until all vertices are in the tree.

The time complexity of Prim’s algorithm depends on the data structures used for the graph and for ordering the edges by weight, which can be done using a priority queue. Table 2 shows the typical choices [33,34], where *V* and *E* are the vertices and the edges of the graph respectively.

**Example for a Possible Application to WSN**: MST can be used to solve the problem of finding the best routes to take when a single source sensor node wants to broadcast a message to a group of sensor nodes (sinks) in the network where each non-source node is both a receiving node and a relaying node. If the (directed) edges’ costs represent the energy that need to be expended on the link, the MST would give us the solution of how to broadcast a message to a set of nodes with the minimum overall energy.

#### 4.2.7. Minimum Cost Network Flow Problems

The transportation, assignment, transshipment, shortest path and maximum flow problems are all special cases of the minimum-cost network flow problem (MCNFP) as shown in Figure 3. Any MCNFP can be solved by a generalization of the transportation simplex called the *network simplex*. To define an MCNFP, let:

- $x_{ij}$: represents the number of units of flow sent from node *i* to node *j* through arc (i,j).
- $b_{i}$: represents the net supply (outflow-inflow) at node *i*.
- $c_{ij}$: be cost of transporting 1 unit of flow from node *i* to node *j* via arc (i,j).
- $L_{ij}$: be the lower bound on flow through arc (i,j), if there is not lower bound $L_{ij}=0$.
- $U_{ij}$: be the upper bound on flow through arc (i,j), if there is not lower bound $U_{ij}=\infty$.

Then the MCNFP may be written as:

(5a) $$min\;\;\sum_{all\;arcs}c_{ij}x_{ij}$$

s.t.

(5b) $$\sum_{j}x_{ij}-\sum_{k}x_{ki}=b_{i}\;\;\forall \;i$$

(5c) $$L_{ij}\leq x_{ij}\leq U_{ij}\;\;\forall \;arcs$$

Constraints (5b) stipulate that the net flow out of node *i* must be equal to $b_{i}$. Constraints (5b) are referred to as the flow balance equations for the network. Constraints (5c) ensure that the flow through the arc satisfies the arc capacity restrictions. This formulation can be used to model all of the special cases of the MCNFP explained earlier.

Since this is a linear program, it can be solved using the simplex method. The *Network Simplex Method* is a special implementation of the simplex method which makes use of the network structure to significantly streamline the computational effort. For a good reference explaining how this algorithm works and other variants of it like *dual network simplex*, *Two-Phase Network Simplex Method* and *One-Phase Primal-Dual Network Simplex Method* the we suggest the lecture slide set in [35] for the reader. It is worth noting that if all the parameters of the NFP problem in Equation (2) are integer, then the number of pivots taken by the network simplex would be *pseudopolynomial* in the network size.

## 5. Fundamentals of Nonlinear Optimization

**Figure 9:**
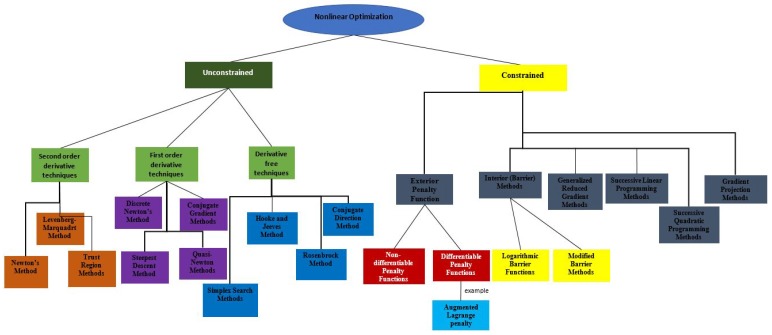
Taxonomy of nonlinear programming (NLP) optimization methods.

This section provides the theoretical discussion on nonlinear programs and their optimality conditions. It forms the theoretical basis of the development of several algorithms for nonlinear optimization. These techniques are illustrated Figure 9, which is a diagram for the taxonomy of those techniques.

A nonlinear programming (NLP) problem deals with the search of a minimum of a function $f\left(x\right)$ of *n* real variables $x=\left(x_{1},x_{2},\ldots ,x_{n}\right)\in X\subseteq R^{n}$ subject to the set of equality constraints $h\left(x\right)=0$ ($h_{i}\left(x\right)=0$, i=1,2,…,m), and a set of inequality constraints $g\left(x\right)\leq 0$ ($g_{i}\left(x\right)\leq 0,\;j=1,2,\ldots ,p$) and is denoted as:

(6a) $$min\;f\left(x\right)$$

s.t.

(6b) $$\begin{array}{c} h\left(x\right)=0 \end{array}$$

(6c) $$\begin{array}{c} g\left(x\right)\leq 0 \end{array}$$

(6d) $$\begin{array}{c} x\in X\subseteq R^{n} \end{array}$$

If any of the functions in Equation (6) is nonlinear, then Equation (6) is an NLP. For the sake of our discussion, we assume that those functions are continuous and twice differentiable.

**Figure 10:**
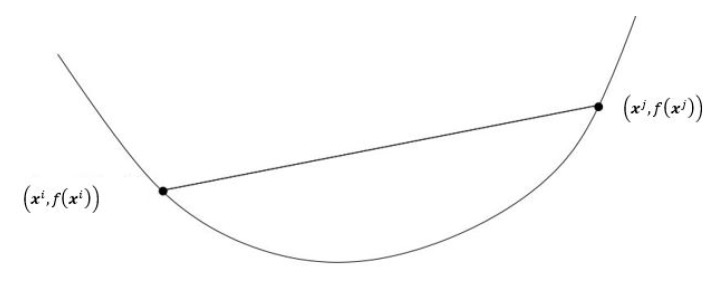
Graph of a convex function. The chord (i.e., line segment) between any two points on the graph lies above the graph.

**Figure 11:**
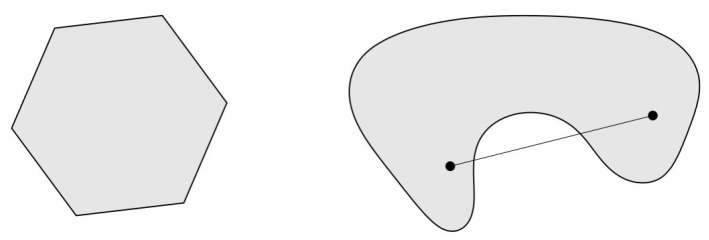
An example of convex and nonconvex sets. *Left*. The hexagon, which includes its boundary (shown darker), is convex. *Right*. The kidney shaped set is not convex, since the line segment between the two points in the set shown as dots is not contained in the set.

We list the following important definitions that are commonly referred to in any discussion on NLPs:

1. **Feasible points**: a point x∈X satisfying all the constraints in Equation (6) is a feasible point and is defined as
  $$F=\left\{x\in X\subseteq R^{n}:h\left(x\right)=0,g\left(x\right)\leq 0\right\}$$
2. **Active, inactive constraints**: An inequality constraint $g_{j}\left(x\right)$ is called active at a feasible point $\bar{x}\in X$ if $g_{j}\left(x\right)=0$, and is called inactive at a feasible point $\bar{x}\in X$ if $g_{j}\left(x\right)<0$. The constraints that are active at a feasible point $\bar{x}$ restrict the feasibility domain while the inactive constraints do not impose any restrictions on the feasibility in the neighborhood of $\bar{x}$, defined as a ball of radius ϵ around $\bar{x}$, $B_{\epsilon }\left(\bar{x}\right)$.
3. **Local minimum:** $x^{*}\in F$ is a local minimum of Equation (6) if there exists a ball of radius ϵ around $x^{*}$, $B_{\epsilon }\left(\bar{x}\right)$:
  $$f\left(x^{*}\right)\leq f\left(x\right)\;\;\;\forall \;\;x\in B_{\epsilon }\left(x^{*}\right)\cap F$$
4. **Global minimum:** $x^{*}\in F$ is a global minimum of Equation (6) if
  $$f\left(x^{*}\right)\leq f\left(x\right)\;\;\;\forall \;\;x\in F$$
5. **Feasible direction vector**: Let a feasible point $\bar{x}\in F$. Then any point x in a ball of radius ϵ around $\bar{x}$ whaich can be written as $\bar{x}+d$ is a nonzero vector if and only if $x\neq \bar{x}$. A vector d≠0 is called a feasible direction vector from $\bar{x}$, if there exists a ball of radius ϵ:
  $$\left(\bar{x}+\lambda d\right)\in B_{\epsilon }\left(\bar{x}\right)\cap F\;\;\;\;\;\;\forall \;\;\;0\leq \lambda \leq \frac{\epsilon }{∥d∥}$$
  The set of feasible directions vectors d≠0 from $\bar{x}$ is called the *cone* of feasible directions of *F* at $\bar{x}$. An important remark is that if $\bar{x}$ is a local minimum of Equation (6) and d is a feasible direction vector from $\bar{x}$, then for sufficiently small λ we must have
  $$f\left(\bar{x}\right)\leq f\left(\bar{x}+\lambda d\right).$$
6. An **improving feasible vector** A feasible direction vector d≠0 at $\bar{x}$ is called an improving feasible direction vector if:
  $$f\left(\bar{x}+\lambda d\right)<f\left(\bar{x}\right)\;\;\;\forall \;\;0\leq \lambda \leq \frac{\epsilon }{∥d∥}$$
  An important remark is that for d≠0 and $d^{T}\nabla f\left(\bar{x}\right)<0$, d is an improving feasible direction vector at $\bar{x}$.
7. **Convex function** is a function $f\left(x\right)$ that satisfies:
  $$f\left(\lambda x^{i}+\left(1-\lambda \right)x^{j}\right)\leq \lambda f\left(x^{i}\right)+\left(1-\lambda \right)f\left(x^{j}\right)$$
  where 0≤λ≤1, this is illustrated in Figure 10. A **concave function** is a function $f\left(x\right)$ that satisfies:
  $$f\left(\lambda x^{i}+\left(1-\lambda \right)x^{j}\right)\geq \lambda f\left(x^{i}\right)+\left(1-\lambda \right)f\left(x^{j}\right)$$
  Strictly convex or concave functions are ones that *strictly* satisfy the inequalities (7) and (7) respectively.
8. **Convex set**: A set S is convex, if and only if a line segment connecting any two points $x^{i},x^{j}\in S$ falls entirely in the set S. This is illustrated in Figure 11.
9. A **convex NLP** is a minimization problem that consists of a convex objective function and a convex feasible set of solutions (feasible regions). A **concave NLP**, is a maximization problem in which the objective function is concave, and the feasible region is a convex set of feasible solutions.

It is worth saying that for a convex (or concave) NLP, every local optimum solution is also globally optimum. Also, another point that is worth mentioning about convex NLPs, is that a sufficient condition for having a convex region is that all the constraint functions $g_{i}\left(x\right)$ be convex functions. One important aspect related to the convexity of multivariate functions is its *Hessian Matrix*$\nabla^{2}f\left(x\right)$ of the function. A convex function must yield a *positive semi-definite* Hessian matrix, i.e., $u^{T}\nabla^{2}f\left(x\right)u\geq 0\;\;\forall u\in R^{n}$. One final word before discussing the Lagrangian function in the following subsection, is that for any local critical point of a function, be it a minimum, maximum or a saddle point, the gradient of the function is necessarily a zero vector, i.e., $\nabla f\left(x\right)=0$.

### 5.1. Lagrange Functions and Multipliers

One important idea in obtaining necessary and sufficient conditions for NLP optimality is to transform a constrained NLP into an unconstrained one. One extremely important and popular transformation involves the use of the auxiliary function called the Lagrangian function and is defined as:

(7) $$L\left(x,\lambda ,\mu \right)=f\left(x\right)+\lambda^{T}h\left(x\right)+\mu^{T}g\left(x\right),\;\;\;\;\;\mu \geq 0$$

where $\lambda^{T}=\left(\lambda_{1},\lambda_{2},\ldots ,\lambda_{m}\right)$ and $\mu^{T}=\left(\mu_{1},\mu_{2},\ldots ,\mu_{p}\right)$ are the Lagrange multipliers associated with the equality and inequality constraints, respectively. The multipliers associated with the equality constraints are unrestricted in sign, while those associated with ≤ inequality constraints are non-negative. The Lagrangian multipliers have a similar interpretation of dual variables in linear programming, and that’s why it is important to understand duality of LPs first. Nonlinear duality is introduced in Section 5.3.

### 5.2. Karush-Kuhn-Tucker (KKT) Conditions

The KKT conditions are widely known conditions that are necessarily satisfied at any feasible solution $\bar{x}$ for Equation (6) that is also a critical point in the objective function (i.e., $\nabla f\left(x\right)=0$), given certain *constraint qualifications* are satisfied. Constraint qualifications can be any of *Slater’s conditions*, *linear independence* of the gradients of active and equality constraints, *Karush-Tucker* constraint qualifications and *weak reverse convex* constraint qualification [30]. If the feasible region is a convex set, and the objective function we are trying to minimize is convex (or maximize a concave function), then the KKT conditions are sufficient conditions for **global** optimality. It is only for very simple problems, usually in an impractical sense, that we could use KKT conditions to solve NLPs. However, devising solution algorithms for solving large NLPs, rely on the insights that are provided by KKT conditions. The following set of equations and inequalities are the KKT conditions:

(8a) $$\nabla f\left(\bar{x}\right)+\lambda^{T}\nabla h\left(\bar{x}\right)+\mu^{T}\nabla g\left(\bar{x}\right)=0$$

(8b) $$h\left(\bar{x}\right)=0$$

(8c) $$g\left(\bar{x}\right)\leq 0$$

(8d) $$\mu^{T}g\left(\bar{x}\right)=0$$

(8e) μ≥0

The conditions in Equation (8) become sufficient for **local** optimality if convexity is satisfied for the objective functions and constraints within a ball of radius ϵ around $\bar{x}$. In other words, a sufficient condition for local optimality would be as follows: if for every non-zero vector $u^{T}\nabla h_{i}\left(\bar{x}\right)=0\;\;\forall \;i$ and $u^{T}\nabla g_{j}\left(\bar{x}\right)=0\;\;\forall \;j\in J\equiv \left\{j:g_{j}\left(\bar{x}\right)\;=\;0\;\right\}$, then,

$$u^{T}\nabla^{2}L\left(\bar{x},\bar{\lambda },\bar{\mu }\right)u\geq 0$$

guarantees a local optimum solution to be $\bar{x}$.

#### 5.2.1. Geometric Interpretation of KKT Necessary Conditions

The KKT conditions show the relation between the gradient of the objective function and the gradients of all the constraint functions. It shows that at a critical point $\bar{x}$, the gradient of the objective function must be a linear combination of the gradients of all the constraints of the problem, where the dual vectors μ and λ represent the weights. The complementary slackness conditions in Equation (8d) enforces the Lagrangian multipliers of the inactive constraints to take zero values. As a result the vector $\lambda^{T}\nabla h\left(\bar{x}\right)+\mu^{T}\nabla g\left(\bar{x}\right)$ belongs to the cone of gradients of the active constraints (i.e., equalities and active inequalities). The geometrical interpretation of the gradient KKT conditions is that $-\nabla f\left(\bar{x}\right)$ belongs to the cone of the gradients of the active constraints at the feasible solutions $\bar{x}$. If $-\nabla f\left(\bar{x}\right)$ lies outside the cone of the gradients of the active constraints, then $\bar{x}$ is not a KKT point.

### 5.3. Duality for Nonlinear Programs

The concept of non-linear duality is one the key things that many solution techniques for different classes of NLPs, use in the core of their operation. First, we define the *Lagrangian dual function* which is given by:

(9) $$D\left(\lambda ,\mu \right)=\underset{x}{min}\left(L\left(x,\lambda ,\mu \right)\right)$$

The dual problem connected to the NLP in Equation (6) is,

(10) $$\underset{\lambda ,\mu }{max}\;\;D\left(\lambda ,\mu \right),\;\;\;\;\lambda \geq 0$$

and finds best lower bound on the optimal objective function value ($p^{*}$) of Equation (6), obtained from Lagrange dual function. This is a convex non-differentiable optimization problem with optimal value ($d^{*}$), where $\left(\lambda ,\mu \right)$ are dual feasible if λ≥0 and $\left(\lambda ,\mu \right)\in \mathrm{dom}\;\left(D\right)=\left\{\left(\lambda ,\mu \right)|D\left(\lambda ,\mu \right)>-\infty \right\}$, and $\mathrm{dom}\left(.\right)$ represents the function’s domain.

As in linear duality, non-linear duality has the *weak duality* property where the optimal objective value of the dual problem is a lower bound on the primal (if it is a minimization problem), i.e., $d^{*}\leq p^{*}$. If the primal problem is unbounded from below, i.e., $p^{*}=-\infty$, we must have $d^{*}=-\infty$ which means the dual is infeasible. If the dual problem is unbounded from above so that $d^{*}=\infty$, then the primal is infeasible and takes the value *∞*. Note that the primal and dual objective functions in this discussion are considered to have infinitely large values outside their domains to be [26]. If the $d^{*}=p^{*}$ holds, i.e., the optimal duality gap is zero, the *strong duality* holds. This means that the best bound that can obtained from the Lagrangian dual functions is tight. This happens usually if Equation (6) is convex. Note that for a non-linear optimization problem to be convex in the presence of equality constraints, these constraints must be linear. If the constraints satisfy at least one *constraint qualification condition*, then strong duality holds.

## 6. Decomposition Methods

Decomposition is a general approach to solving a problem by breaking it up into smaller ones and solving each of the smaller ones separately, either in parallel or sequentially. When it is done sequentially, the advantage comes from the fact that problem complexity grows more than linearly. Problems for which decomposition works in one step are called (block) separable, or trivially parallelizable. As an example of such a problem, suppose the variable x can be partitioned into subvectors $x_{1},\ldots ,x_{k}$ the objective is a sum of functions of $x_{i}$, and each constraint involves only variables from one of the subvectors $x_{i}$. Then evidently we can solve each problem involving $x_{i}$ separately (and in parallel), and then re-assemble the solution x. There are many situations in which there is some connection between the subvectors, so the problems cannot be solved independently. For these cases there are techniques that solve the overall problem by iteratively solving a sequence of smaller *subproblems* and a *master problem*. Basically there are two broad classes of decomposition techniques, these are the *primal decomposition* and the *dual decomposition* techniques, we suggest S. Boyd notes [36] on decomposition techniques. The subgradient methods are among the popular techniques used to solve the *master problem*, for which we refer the reader to the notes of S.Boyd [37] on that topic. It is worth mentioning that decomposition schemes can be used for LPs, NLPs, MILPs and MINLPs.

Decomposition techniques can be used to solve a problem that has the following form:

(11a) $$\underset{x}{min}\;\;f_{1}\left(x_{1}\right)+f_{2}\left(x_{2}\right)$$

s.t.

(11b) $$x_{1}\in X_{1},\;x_{2}\in X_{2}$$

(11c) $$g_{1}\left(x_{1}\right)+g_{2}\left(x_{2}\right)\leq 0$$

where $X_{1}$ and $X_{2}$ are feasible regions of two separate subproblems, which may be described by linear equalities and convex inequalities. The subproblems are connected by a set of *m* coupling constraints that involve both $x_{1}$ and $x_{2}$.

### 6.1. Primal Decomposition

To use primal decomposition to solve a problem with the structure of Equation (11), a variable $y\in R^{m}$ can be introduced, which represents the amount of resources allocated to the first subproblem. Obviously, −y is allocated in the second subproblem. Our first subproblem is then given by:

(12a) $$\underset{x_{1}}{min}\;\;f_{1}\left(x_{1}\right)$$

s.t.

(12b) $$x_{1}\in X_{1},\;\;g_{1}\left(x_{1}\right)\leq y$$

and the second subproblem becomes

(13a) $$\underset{x_{2}}{min}\;\;f_{2}\left(x_{2}\right)$$

(13b) $$x_{2}\in X_{2},\;\;g_{2}\left(x_{2}\right)\leq -y$$

Let $\Psi_{1}\left(y\right)$ and $\Psi_{2}\left(y\right)$ denote the optimal values of the subproblems (12) and (13), respectively. The original problem becomes equivalent to the master problem of:

(14) $$\underset{y}{min}\;\;\Psi =\Psi_{1}\left(y\right)+\Psi_{2}\left(y\right)$$

and the subproblems can be solved separately when y is fixed.

A subgradient for the optimal value of each subproblem from an optimal dual multiplier associated with the coupling constraint can be obtained. For e.g., if for the first subproblem the optimal solution is $\Psi_{1}\left({\bar{y}}_{1}\right)$, and ${\bar{y}}_{1}\in \mathrm{dom}\;\left(\Psi_{1}\right)$. If $\lambda \left(y\right)$ is an optimal dual vector of variables associated to the constraint set $g_{1}\left(x_{1}\right)\leq \bar{y}$, then $-\lambda \left(y\right)$ is a subgradient of $\Psi_{1}$ at ${\bar{y}}_{1}$. Therefore, to find a subgradient of Ψ, we solve the two subproblems (12) and (13), to find the optimal solutions $x_{1}$ and $x_{2}$ as well as the dual variable vectors $\lambda_{1}$ and $\lambda_{2}$ associated with the constraint sets $g_{1}\left(x_{1}\right)\leq y$ and $g_{2}\left(x_{2}\right)\leq -y$, respectively. In this case a subgradient would be $\left(\lambda_{2}-\lambda_{1}\right)$. If $\bar{y}\notin \mathrm{dom}\;\Psi$, a cutting plane can be generated to separate $\bar{y}$ from domΨ, for use in solving the master problem.

A primal decomposition algorithm can be summarized as follows:

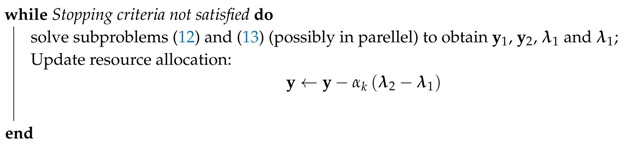
(15)
where $\alpha_{k}$ is an appropriate step size. At every step of this scheme, we have feasible solutions to the original problem.

### 6.2. Dual Decomposition

In dual decomposition, the partial Lagrangian is first obtained:

(16) $$\begin{array}{cc} L\left(x_{1},x_{2},\lambda \right) & =f_{1}\left(x_{1}\right)+f_{2}\left(x_{2}\right)+\lambda^{T}\left(g_{1}\left(x_{1}\right)+g_{2}\left(x_{2}\right)\right)\\ & =\left(f_{1}\left(x_{1}\right)+\lambda^{T}g_{1}\left(x_{1}\right)\right)+\left(f_{2}\left(x_{2}\right)+\lambda^{T}g_{2}\left(x_{2}\right)\right) \end{array}$$

which is separable in $x_{1}$ and $x_{2}$. Given the dual variable vector λ, the *master problem* then becomes:

(17) $$\underset{\lambda }{max}\;\;\Phi \left(\lambda \right)=\Phi_{1}\left(\lambda \right)+\Phi_{2}\left(\lambda \right)$$

To find $\Phi_{1}\left(\lambda \right)$, we solve:

(18a) $$\underset{x_{1}}{min}\;\;\left(f_{1}\left(x_{1}\right)+\lambda^{T}g_{1}\left(x_{1}\right)\right)$$

s.t.

(18b) $$x_{1}\in X_{1}$$

and to find $\Phi_{2}\left(\lambda \right)$, we solve

(19a) $$\underset{x_{2}}{min}\;\;\left(f_{2}\left(x_{2}\right)+\lambda^{T}g_{2}\left(x_{2}\right)\right)$$

s.t.

(19b) $$x_{2}\in X_{2}$$

A subgradient for $-\Phi_{1}$ at λ is $g_{1}\left({\bar{x}}_{1}\right)$ is any feasible solution to Equation (18). To find a subgradient of Phi, both subproblems need to be solved to get their optimal solutions $x_{1}^{*}$ and $x_{2}^{*}$. A subgradient of −Phi is $g_{1}\left(x_{1}^{*}\right)+g_{2}\left(x_{2}^{*}\right)$. The master problem updates the dual variable vector λ based on this subgradient, in a *projected subgradient method* [37]. The framework of the dual decomposition is as follows:

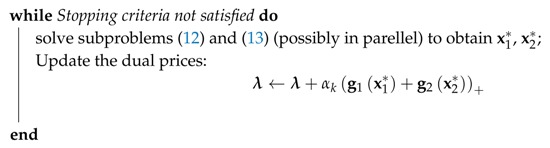
(20)

Each iteration gives a lower bound on the optimal solution objective value. The iterates are not necessarily feasible, however, feasible solutions can be obtained using certain techniques, whose objective function value becomes an upper bound on the optimal solution.

## Part III Optimization in WSN Design Problems

In this part of the paper, we present 14 design problems in WSNs, half of them consider the routing aspect as the taxonomy in Figure 1 shows. We summarize the design problems and the corresponding optimization techniques used in Table 3 whose columns show:

1. the design problem,
2. the initial optimization problem formulations,
3. the design objective,
4. the centralized/distributed possible algorithmic implementation to solve the initial formulations,
5. any reformulations performed,
6. solution algorithms that were proposed,
7. whether the proposed algorithms are distributed or centralized,
8. the nature of the solution that could be obtained, whether it is suboptimal or global optimal,
9. the convergence speed or computational complexity of the solution algorithms.

As the taxonomy in Table 3 shows, almost all the problems considered in this paper had initial formulations that could only be solved in centralized fashion. However using some good reformulation and solution techniques like the dual decomposition or problem specific heuristics, the problems could be solved in a distributed fashion. Therefore, as demonstrated throughout the paper, formulation techniques are always the key to the algorithms to implement. The speed of convergence of these algorithms and whether they can be implemented in distributed schemes, are consequences of the type of formulation the problem gets reduced to. In the rest of this part (part III), each section is dedicated to each design problem, which is chosen from a paper in the literature. Once again, we would like to emphasize that the selection of the different papers is done so that a wide range of applications and design aspects for which optimization techniques are used, are presented to the reader.

## 7. Routing for Multi-hop WSNs with a Single Immobile Sink

### 7.1. System Model and Design Objectives

A multi-hop wireless sensor network was considered in [2] that focuses on computing multi-hop routes from each node to a single immobile sink such that the network lifetime is maximized. The lifetime in [2] was defined as the time at which the first node runs out of energy. Each node can generate information due to its sensing capabilities and relay packets from other nodes to the sink node. The nodes’ battery energies are limited and adjustments of transmission powers for each node is possible depending on the distance between nodes.

### 7.2. Initial Optimization Problem Formulation

The initial formulation given in [2] is a max-min non-linear program where the objective function is to maximize the minimum of all lifetimes across the nodes in the network. The life time of each node is defined as the quotient of the initial battery energy of the node to the sum of expended energies on each of the node’s outgoing flows. A brief explanation of the formulation is given as follows:

- Decision variables: Continuous flow variables for all links in the network. Each of these variables has a lower bound of zero and an upper bound of the link’s capacity.
- Objective function: Maximizes the minimum life time of all nodes given as $T_{i}\left(r\right)=\frac{B_{i}}{\sum_{j\in N_{i}}^{}E_{ij}r_{ij}}$ where $B_{i}$ is the initial battery energy for node *i*, $E_{ij}$ is the energy spent per bit to transmit data from node *i* to node *j* on a direct link, $N_{i}$ is the set of neighbor nodes that have direct arc connections with *i*, and $r_{ij}$ is the flow decision variable for link $\left(i,j\right)$.
- Constraint set: Is a linear equality set of conservation of flow constraints for all the nodes in the network. They simply state that the difference between the outgoing flows from each node and its incoming flows should strictly be equal to the data generated by the node itself.

To make the problem easier, the minimum term in the objective function is replaced by an auxiliary variable, that is $T=\underset{i}{min}T_{i}\left(r\right)$. In order to guarantee that the new objective function has an equal value to the previous one, an upper bound constraint is enforced on the new variable *T* that represents the energy conservation constraint for each node, that is $T\leq \frac{B_{i}}{\sum_{j\in N_{i}}^{}E_{ij}r_{ij}}$ for all nodes. This makes the problem a bilinear quadratically constrained program.

### 7.3. Reformulation

The problem in [2] is linearized (i.e., transformed to a linear program) by a simple reformulation trick in which the reciprocal of the lifetime decision variable *T* is replaced by another variable *q* which becomes the new objective function and minimized instead (i.e., minq=1/T). This directly replaces the quadratic constraint set $T\leq \frac{B_{i}}{\sum_{j\in N_{i}}^{}E_{ij}r_{ij}}$ with linear ones $\sum_{j\in N_{i}}^{}E_{ij}r_{ij}\leq qB_{i}$.

### 7.4. Solution Methods

Two solution methods were provided in [2]:

#### 7.4.1. A partially Distributed Algorithm

In which the Lagrangian for the objective function is obtained. The dual function is the minimum Lagrangian function where the minimizers are the primal variables of the problem *q* and all $r_{ij}$. The primal decision variables *q* and $r_{ij}$ appear in separate additive terms in the Lagrangian and hence the dual function can be evaluated separately in r (the vector of flow variables) and *q*.

The primal objective function, *q*, is modified to an equivalent strictly convex function $q^{2}$ plus a strictly convex regularization term $\epsilon \sum_{i\in V}^{}\sum_{j\in N_{i}}^{}r_{ij}^{2}$. Also a loose upper bound is imposed on the variable so that all decision variables have bound constraints that form a bounded polyhedron. These two modifications ensure that the dual function is differentiable and hence enables the use of the subgradient algorithm with guarantee that it converges to the solution of the strictly convexified primal problem. The dual function for the strictly convexified problem is still separable in the primal variables *q* and each of $r_{ij}$. In each iteration *k* of the subgradient algorithm, $q^{k}$ and $r_{ij}^{k}$ are obtained by solving box constrained convex quadratic single variable optimization problems. The obtained values of the primal variables are used to calculate the values of the dual variables from the next iteration by the subgradient formulas.

At a given iteration *k* in this algorithm, the values of the dual variables needed to solve for the flow variables in each iteration *k* are locally available to a node *i*. This means that optimum rate decision variables can be obtained separately at each node without any communication overhead between the nodes. The calculation of *q* however, needs all the values dual variable values in iteration *k*,$\lambda_{k}$, from all the nodes in the network to be transmitted to the node responsible for the computation of $q^{k}$ in every iteration *k*. The values of $q^{k}$ need to be broadcasted to all the nodes as they are needed for the subgradient calculation.

For the *i*th element of the subgradient to be computed at node *i* for iteration *k*, it needs the rate variables $r_{ji}^{k}$ for all its neighbors in the set $N_{i}$ to be transmitted to it. Therefore, each node has to broadcast its $r_{ij}^{k}$ values to its neighbors. Then, using those, the value of $q^{k}$ received from a broadcast and the locally calculated $r_{ij}^{k}$, the *i*th element of the subgradient gets calculated at node *i*. The values of the new dual variables are calculated locally at each node.

Since every node contributes in the computation of the primal variables, dual variables and the subgradient at each iteration, the algorithm is therefore like a distributed one. However, the algorithm is not fully distributed since at iteration *k*, node *i* still needs other calculated variables from other nodes. Another algorithm was proposed in [2] that is fully distributed.

#### 7.4.2. A fully Distributed Algorithm

The linear program is transformed into a strictly convex quadratic optimization problem by introducing a separate variable *q* for each node *i*. Then, instead of maximizing the primal objective function in a single variable $q^{2}$ as in the problem, the sum of $q_{i}^{2}$ is maximized. By enforcing an equality constraint $q_{i}=q_{j},\forall i\in V,\forall j\in N_{i}$ (where *V* is the set of nodes and $N_{i}$ is the set of neighbors of *i*) which guarantees that for any feasible solution the objective function is just $\left|V\right|q^{2}$ which yields the same set of feasible solutions and the same optimal solution.

This change enables the dual problem to be decomposed to separate node local problems which each node can solve independently with only the exchange of dual variables with its neighbors.

### 7.5. Important Results

**Figure 12:**
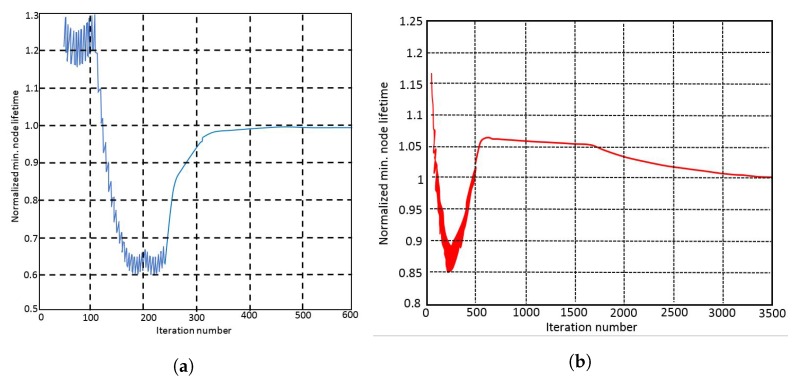
Number of subgradient iterations needed for convergence by the partial and fully distributed schemes proposed in [2]. (**a**) Partially distributed scheme; (**b**) Full distributed scheme.

The number of iterations required for convergence of the partially distributed algorithm in [2] is around 600 iterations while the number for the fully distributed algorithms is around 3500 iterations as shown in Figure 12. Therefore, quicker convergence is achieved in partial distribution at the expense of higher communication overhead due to larger number of variable exchanges every iteration of the algorithm as compared to the fully distributed algorithm. On the other hand the fully distributed algorithm has variable exchanges between each node and its neighbors only and hence has lower communication overhead.

### 7.6. Discussions of the Results

The fully distributed algorithm requires less communication overhead per iteration, but more than five times the number of iterations that the partially distributed algorithm requires to converge and stop. The aggregate overhead and computational effort over all the iterations were not shown in the results in [2]. It could still be that the partially distributed algorithm requires a lower total communication overhead and computational effort since it requires lower iterations to converge. In this case the fully distributed algorithm would loose its appeal. Therefore, both algorithms should be evaluated for the total computational effort and the total required communication overhead for all the iterations in order to make an accurate comparison.

## 8. Routing in a delay-tolerant WSN to a Single Mobile Sink

### 8.1. System Model and Design Objectives

A WSN with a mobile sink was considered in [3]. Each node can postpone data transmission until the sink is at the most favorable position to extend the lifetime of the network. The problem is to find how long the sink should stay at potential stops and how buffered data could be routed to the sink when it stops taking into account a maximum delay toleration. A distributed algorithm was used in which the problem is decomposed to smaller decision problems and each can be solved by a sensor node. Only local information from the neighbors is needed by each node.

### 8.2. Initial Optimization Problem Formulation

The optimization problem was initially formulated as a quadratically constrained program QCP. The following are the details of the formulation:

- Decision variables: are all continuous and are classified as follows,
  1. The actual decision variable sets are the time the sink stays at each location l∈L within each tour, these were denoted by $t_{l}$, and the data rate from node *i* to node *j* while the sink is at position *l*, that were given as $a_{ij}^{\left(l\right)}$. These two sets were replaced by the variables $x_{ij}^{\left(l\right)}$, which represent the data flow between two nodes *i* and *j* with the sink at position *l*, through the relation $x_{ij}^{\left(l\right)}=t_{l}a_{ij}^{\left(l\right)}$.
  2. $y_{i}^{\left(l\right)}$ represents the the amount of buffered data at node *i* just as the sink leaves location *l*.
  3. *T* represents the number of cycles the mobile sink makes.
- Objective function: Maximize the number of cycles the mobile sink makes i.e., $\underset{T,x_{ij}^{\left(l\right)},y_{ij}^{\left(l\right)}}{max}T$. This is a linear objective function in one continuous variable.
- Constraint Sets: The interpretation of the constraint sets is as given below,
  1. Linear equality constraints that combine the transmission flows and buffered data for all possible sink locations to enforce conservation of flow constraints which guarantee that the total incoming flows for node *i* plus the buffered data is equal to the outgoing flows.
  2. Non-negativity constraints on all variables that represent the flows, the buffered data and the number of rounds made by the mobile sink.
  3. A bi-linear quadratic constraint set that guarantees that all the energy expended due to data transmission on the links for all possible sink positions. It was given by $\left(\sum_{l=1}^{L}\sum_{j\in N_{l}\left(i\right)}e_{ij}^{\left(l\right)}x_{ij}^{\left(l\right)}\right)T\leq E_{i},\;\;\forall i\in N$ where $e_{ij}^{\left(l\right)}$ is the energy spent per unit data on the link $\left(i,j\right)$ when the sink is at position *l* and $E_{i}$ is the available energy for node *i* and $E_{i}$ is the available battery energy for node *i*.

### 8.3. Comments on the Problem Formulation

In [3] a replacement of decision variables was done by using $x_{ij}^{\left(l\right)}=t_{l}a_{ij}^{\left(l\right)}$ and solving for $x_{ij}^{\left(l\right)}$ instead. The obtained values are used to obtain $a_{ij}^{\left(l\right)}$ by assigning arbitrary values to the original variables $t_{l}$ that satisfy $\sum_{l=1}^{L}t_{l}\leq D$. This assumes that the data rate $a_{ij}^{\left(l\right)}$ is unbounded which is not practical. For the locations which do not get visited, $t_{l}$ will have to be assigned zero values which means infinite data rate $a_{ij}^{\left(l\right)}$.

### 8.4. Reformulation for the Optimization Problem Formulation

The problem was reformulated to a linear program by minimizing the reciprocal of the number of sink cycles and substituting that reciprocal with another continuous variable z=1/T. The bi-linear constraint now becomes $\left(\sum_{l=1}^{L}\sum_{j\in N_{l}\left(i\right)}e_{ij}^{\left(l\right)}x_{ij}^{\left(l\right)}\right)\leq zE_{i},\;\;\forall i\in N$ in the new formulation.

### 8.5. Solution Method

Lagrangian relaxation was used to dualize the set of flow equality constraints. The Lagrangian dual function is then minimized with respect to the primal variables z,x and y where the vectors x and y comprise of the variables $x_{ij}^{\left(l\right)}$ and $y_{i}^{\left(l\right)}$ respectively. The Lagrangian dual optimization problem minimizes the Lagrangian dual function subject to the energy constraint for each node (which got linearized as mentioned earlier) and decision variable bounds constraints.

The Lagrangian dual optimization problem is decomposed into two subproblems S1 and S2. The subproblem S1 consists of a linear objective function in the primal vector y and decision variable bound constraints on the variables $y_{i}^{\left(l\right)}$. Subproblem S2 consists of a linear objective function in *z* and the vector x. The constraints for S2 are the energy constraints $\left(\sum_{l=1}^{L}\sum_{j\in N_{l}\left(i\right)}e_{ij}^{\left(l\right)}x_{ij}^{\left(l\right)}\right)\leq zE_{i},\;\;\forall i\in N$ and variable bound constraints.

One subproblem was reduced to a linear box constrained problem whose minimum value was obtained in a distributed manner by each node by setting its corresponding buffering variable $y_{i}^{\left(l\right)}$ to their upper bound if their respective coefficient in the objective function of the subproblem is negative and zero otherwise.The second subproblem was reduced to multiple fractional knapsack problems that could be solved separately by each node (hence decentralized approach) in polynomial time. The subgradient algorithm was used to evaluate the values of the dual variables on an iteration by iteration basis.

### 8.6. Important Results

**Figure 13:**
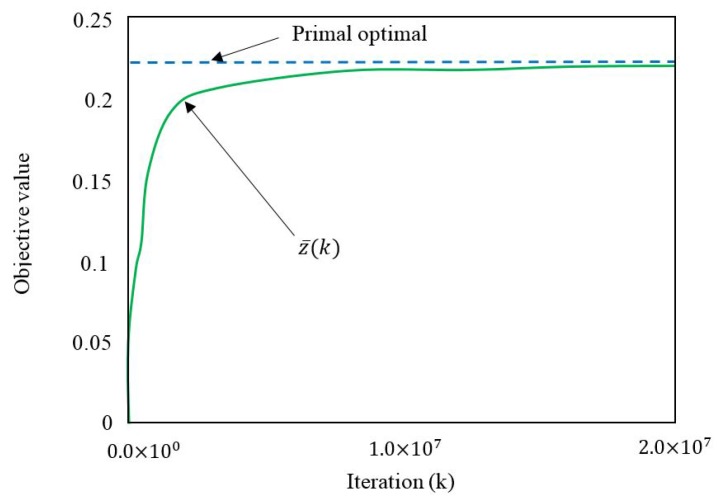
Convergence to the Optimal Solution in [3].

The algorithm was analyzed based on Lyapunov drift and was shown that it converges to the optimal solution in the average long run and that the long-time average of the virtual queues are bounded. Numerically, a good feasible suboptimal solution was obtained after a few hundred iterations (100–200) and the objective value improves monotonically as shown in Figure 13.

## 9. Joint Routing, Power and Bandwidth Allocation in FDMA WSNs

### 9.1. System Model and Design Objectives

In [4] a frequency division multiple access (FDMA) based single-immobile-sink WSN was considered. The objective was to jointly allocate data flows and bandwidth for the network links in order to minimize the total transmission power in the WSN. Flat fading was assumed which makes the link rates dependent only on the power levels and the bandwidth of the link irrespective of frequency dependent gains. Each sensor *i* has a limited total power *P* and a total preallocated bandwidth $W_{i}$. The power and bandwidth allocated to the sensor’s links $l\in O\left(i\right)$ should satisfy $\sum_{l\in O\left(i\right)}^{}p_{l}\leq P$ and $\sum_{l\in O\left(i\right)}w_{l}\leq W_{i}$∀i∈S, where *S* is the set of nodes and $O\left(i\right)$ is the set of the outgoing links of node *i*.

Each node allocates disjoint and continuous frequency bands to its outgoing links. All nodes are assumed to have a maximum communication range, therefore a link between two nodes exists only if both are within the communication range of each other. The flow on a link cannot exceed Shannon ’s capacity of the link which was compactly represented as $c_{l}\left(p_{l},w_{l}\right)=w_{l}log\left(1+\gamma_{l}\frac{p_{l}}{w_{l}}\right)$ where $\gamma_{l}$ is a constant related to the link *l*.

### 9.2. Optimization Problem Formulation

The following are the details of the optimization problem formulated in [4]:

- Decision variables: All the decision variables are continuous non-negative variables. There are three sets, one set of variables is for the power values on the links $p_{l}$, the second is for the flow capacities on the links $f_{l}$ and the third is for the amounts bandwidth spectrum allocated to the links in the network $w_{l}$.
- Objective function: is a linear function in the aggregate link powers i.e., $\sum_{l\in L}^{}p_{l}$ (where L is the set of links).
- Constraint sets: There are four functional constraint sets, these are,
  1. a linear equality constraint set in the flow variables $f_{l}$ for the conservation of data rate flows. These guarantee that for every node the difference between the out-going flows and the sum of the in-going flows is strictly equal to the rate of data generated by each node $t_{i}$.
  2. a convex non-linear constraint set that guarantees that the flows on each link are upper bounded by the Shannon capacity of the link. These constraints are function in both the powers on the links $p_{l}$ and the bandwidths allocated to the links $w_{l}$ and are given as, $f_{l}\leq c_{l}\left(p_{l},w_{l}\right)\;\;\forall l\in L$.
  3. a linear constraint set in the power variables of the links that guarantees that for each node the aggregate transmission power on all its links does not exceed sensor’s battery power, i.e., $\sum_{l\in O\left(i\right)}^{}p_{l}\leq P,\forall i\in S$.
  4. A linear constraint set in the bandwidth variables that guarantee that the sum of bandwidths allocated on all the links of every node does not exceed the nodes’ pre-allocated bandwidth *W*, i.e., $\sum_{l\in O\left(i\right)}^{}w_{l}\leq W,\forall i\in S$.

### 9.3. Reformulation: Distributed Global Consensus Problem

The objective function and all constraints but the flow conservation constraint set can be considered an independent local problem for each node to solve. Consensus reformulation is used by introducing local copies ${\hat{f}}_{il}$ of the associated global flow variables for each node.The local variables were interpreted in [4] as the node’s opinion about the corresponding global flow variables. By carrying out the following modifications to the formulation, the problem becomes a global consensus problem:

1. ${\hat{f}}_{il}\leq c_{l}\left(p_{l},w_{l}\right)\;\;\forall l\in L,\;\;\forall i\in S,\;l\in O\left(i\right)$
2. $\sum_{l\in L\left(i\right)}a_{il}{\hat{f}}_{il}=r_{i,}\;\forall i\in S$
3. ${\hat{f}}_{il}=f_{l}\;\;\forall i\in S,l\in L\left(i\right)$, where $L\left(i\right)$ is the set of all links, incoming and outgoing from node *i*, represent the consensus constraints.

Except for the consensus constraints, the rest of the modified constraints are local to each node. The global consensus problem is compactly given by:

(21) $$minimize\;\sum_{i\in S}^{}g_{i}\left(p_{i},w_{i},{\hat{f}}_{i}\right):{\hat{f}}_{i}=f_{l},\;\;\forall i\in S,\;l\in L\left(i\right)$$

where

(22) $$g_{i}\left(p_{i},w_{i},{\hat{f}}_{i}\right)=\left\{\begin{array}{c} \sum_{l\in O}p_{l}\;\mathrm{if}\;\left(p_{i},w_{i},\hat{f_{i}}\right)\in X_{i}\\ \infty \;\;\;otherwise \end{array}\right.$$

and $p_{i}$, $w_{i}$, $\hat{f_{i}}$ are its power, bandwidth and local flow vectors of variables for each node that must satisfy the local node constraints of total power, bandwidth and flow capacity [4] (i.e., $\left(p_{i},w_{i},\hat{f_{i}}\right)\in X_{i}$). These are similar to the global problem whose formulation was described in the previous section.

### 9.4. Solution Method

The augmented Lagrangian for the problem’s global consensus reformulation is obtained with respect to the consensus constraints. An $L_{2}$ norm penalty term is added to regularize the non-differentiable optimization function so that convergence is possible due to the non-differentiable nature in the objective function $g_{i}\left(p_{i},w_{i},{\hat{f}}_{i}\right)$. The ADMM method consists of a sequence of optimization phases over the primal variables followed by a gradient method that updates the dual variables.

In each node, phase 1 minimizes the augmented Lagrangian over the node local variables ($p_{i},w_{i}$ and $\hat{f}$). The second phase minimizes over the global flow variable ($f_{i}$) for each node *i*. Then, the dual variable corresponding to each link for the node is updated with the constant step size ρ. Phase 1 problem is a quadratic convex optimization problem in the local resource variables and the local flow variables. Interior-point methods were used by the authors to solve it. The phase 2 problem was manipulated algebraically to give the simple form:

(23) $$f_{l}^{k}=\left\{\begin{array}{c} \frac{1}{2}\left({\hat{f}}_{tran\left(l\right)}^{k}+{\hat{f}}_{rec\left(l\right)}^{k}\right),for\;all\;but\;the\;sink\;node\\ {\hat{f}}_{tran\left(l\right)}^{k},for\;the\;sink\;node \end{array}\right.$$

where *k* is the iteration index, $tran\left(l\right)$ and $rec\left(l\right)$ are the transmitting and receiving nodes on the link *l* respectively. Thus, the global flow variables are obtained at each iteration *k* by averaging out the corresponding updated local variables.

The only information that needs to be shared among the nodes are the local flow variables. These have to be broadcasted by each node to its neighbors. The communications overhead therefore depends on the network density, rather than the number of nodes.

### 9.5. A Reference Algorithm

**Figure 14:**
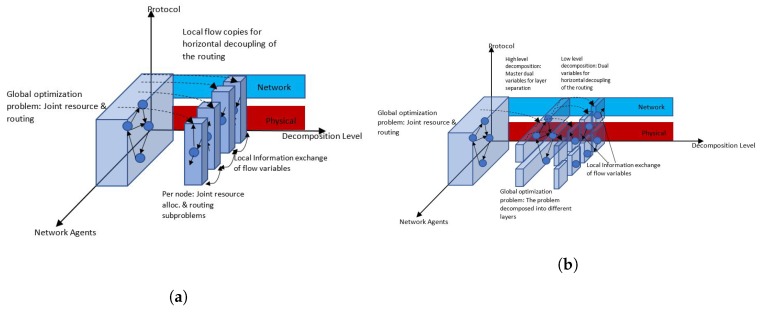
Hierarchical representation of different decomposition steps in [4]. (**a**) Distributed consensus ADMM; (**b**) Dual decomposition approach.

The authors also proposed a dual decomposition approach for the sake of comparison. An augmented Lagrangian approach is obtained for the proximal regularized objective function. Besides the decomposition, across the nodes, the primal problem at each node is decomposed into two separate problems for routing and resource allocation, hence a protocol layer decomposition. The levels of problem decomposition are shown in Figure 14. The projected subgradient method is used to obtain the dual variables at each node for the dual decomposition scheme.

### 9.6. The Obtained Results

**Figure 15:**
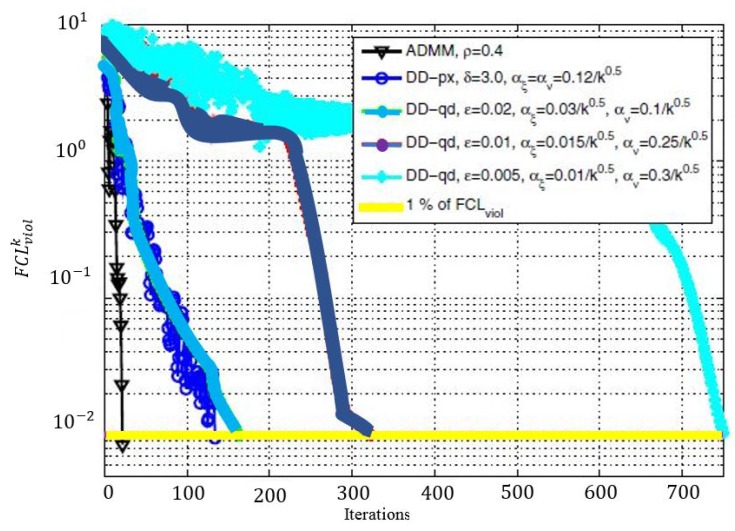
Convergence of the ADMM consensus scheme versus the dual decomposition scheme represented by the flow conservation constraint violation [4].

The authors showed that the consensus optimization using ADMM converged much faster than dual decomposition technique. For a network of eight nodes, the ADMM converged in around 21 iterations while the dual decomposition technique needed at least 133 iterations to converge as Figure 15 illustrates.

## 10. Routing and Energy Allocation in Rechargeable WSNs with Multiple Sources and Destinations

### 10.1. System Model and Design Objectives and Considerations

In [5], a rechargeable WSN whose batteries’ replenishment profile is unknown a priori is considered. Aggregate utility functions for the sensors are to be maximized with low complexity. It was shown that the problem can be formulated as a standard convex optimization problem with energy and routing constraints. However, the solution requires centralized control and full knowledge of replenishment profiles in the future, which may not be available in practice. Therefore, a low-complexity heuristic solution was developed that is asymptotically optimal and can be approximated by a distributed algorithm. The following are the main elements of the system model in [5]:

1. Time slotted system with finite number of slots was considered,
2. the battery of each sensor is assumed to have an infinite rechargeable capacity,
3. multiple sensing sources and multiple destination nodes are considered,
4. utility function that reflects the “satisfaction” of the node is associated with each source node when it transmits at an average data rate ${\hat{x}}^{s}\left(t\right)$ that is equal to the aggregate amount of data from that source to a particular destination over all time slots averaged over the duration of the frame. It is defined generally to be concave monotonically increasing in the average data rate of the source node.

### 10.2. Problem Formulation

A formulation that maximizes the sum of general utilities of all sensor nodes was formulated as a convex NLP. A brief explanation for the formulation is given below:

- Decision variables: All decision variables are continuous variables. There are three sets of decision variables,
  1. $w_{ij}\left(t\right)$ is the amount of data on the outgoing link $\left(i,j\right)$ for time slot *t*,
  2. $x^{s}\left(t\right)$ is the amount of data delivered from source $f_{s}$ to the destination $d_{s}$,
  3. $e_{s}\left(t\right)$ represents the amount of energy expended by a node *s* during slot *t*.
- Objective function: is the sum of individual node utilities where each of these, $U_{s}\left(\frac{1}{T}\sum_{t=1}^{T}x^{s}\left(t\right)\right)$, is a function of the amount of data delivered from source node $f_{s}$ to destination node $d_{s}$ in all *T* time slots over possibly multiple hops and multiple paths. Each utility function is assumed to be a continuous non-linear concave function. The time parameter in [5] is discrete.
- Constraint sets: There are three constraint sets, these are:
  1. non-negative flow constraints on the flow variables $w_{ij}\left(t\right)\geq 0$.
  2. conservation of flow constraints that are linear constraints in the decision variables that represent the flow and amount of data delivered from a source node to a destination node, $w_{ij}\left(t\right)$ and $x^{s}\left(t\right)$, i.e., $\sum_{t=1}^{T}\sum_{j}w_{ij}^{d}-\sum_{t=1}^{T}\sum_{j}w_{ji}^{d}-\sum_{t=1}^{T}\sum_{S:f_{s}=i,d_{s}=d}x^{s}\left(t\right)\geq 0$.
  3. The third ensures that the sum of flows emanating from a node *i* belongs to the set $\Lambda_{i}$ of the different amounts of data in different time slots under a given replenishment profile vector ${\vec{r}}_{i}$. For any data vector in $\Lambda_{i}$, there exists an energy vector $e_{n}$ that achieves that amount of data for a given modulation and coding scheme. The set $\Lambda_{i}$ was proved to be convex in works earlier to [5].

### 10.3. Comments on the Problem Formulation

The following are our comments on the formulation in [5]:

1. The authors assume that the generic objective function, given as the sum of the utility functions of all sensor nodes, is concave without stating why is this expected. We believe the characteristics of a generic objective function should cover a wide range of possible objective functions. So, it would be interesting to know how concavity is expected for most of the objective functions in similar system models.
2. The relation between the amount of data transmitted by a node on a particular time slot and the expended energy for that is not observable in the formulated problem.
3. There were no constraints enforced that take into account the amount of available energy for each node. This may lead to a solution which cannot be satisfied due to lack of energy resources.
4. Since there are no non-negativity constraints on the variables that represent the amount of energy expended by the sensor, it is not clear how this formulation guarantees non negative solutions for the energy variables.
5. The relation between the allocation of energy and the replenishment is not clear in the formulation.
6. The problem is just a generic convex optimization problem so the authors state that standard convex optimization algorithms can be used to solve it. The authors then claim that this is still too complex to solve, even for a known replenishment profile. We believe, however, that a more specific problem (a specific objective and a clear connection between variables) could have more embedded useful structure that if exploited could be solved with lower complexity algorithms.

### 10.4. Solution Method

A heuristic method named DualNet was proposed that obtains an infeasible upper bound and a feasible lower bound and iteratively solves the problem until it converges to the optimal solution infinity. First an upper bound was obtained on the value of the objective function at the optimal solution of the problem after a long period of time (theoretically infinity). The solution that gives the upper bound is obtained by an infeasible energy allocation i.e., energy allocation that is higher than the average replenishment rate. The energy allocation (and hence the routing solution) are the same over all time slots and is more than the average replenishment rate, yielding infeasiblity. Using the energy allocation obtained, a routing sub-problem that is strictly convex and computationally easier than the original problem is obtained because of the decoupling of the time component. This requires solving the problem every time slot.

The lower bound solution is obtained by assigning a feasible energy value in each time slot for each node. The energy assignment for a node is the minimum of either the average harvested energy or the available battery energy (including the instantaneous replenishment for a given time slot). This assignment is done by each node on its own and hence is a distributed energy assignment. Using the energy assignment values the routing subproblem that obtains the lower bound, is again a similar routing subproblem to that of the upper bound subproblem.

Dual decomposition was used to solve the problem which enabled a distributed implementation of the scheme. Each source node solves two problems, one to determine the amount of data to inject in the network at a given time slot *t*, $x_{s}\left(t\right)$, the other subproblem to determine the routes and their flows, $w_{ij}\left(t\right)$. All the nodes that are not sources of data, and only responsible for relaying data over multiple hops, solve the routing problem only. The dual variables are computed using the subgradient algorithm.

### 10.5. Comments and Observations on the Solution Technique

The decoupled routing sub-problem is simpler than the main problem formulation if it is solved for one time slot. However there are no analyses or results provided to show the computational effort required to solve the routing sub-problem *T* times versus solving the original formulated problem once over the time span of a TDMA frame. Therefore, we are not sure which would be better, whether the heuristic scheme is really quicker than optimally solving the original problem which is a convex one.

### 10.6. Comments on the Experiments and Results

**Figure 16:**
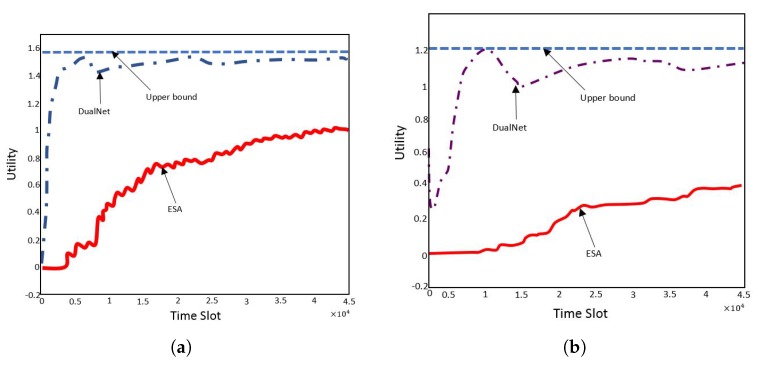
Number of subgradient iterations needed for convergence in the distributed schemes proposed in [5]. (**a**) Utility Performance for Solar Energy Recharging; (**b**) Utility Performance for Wind Energy Recharging.

We have three comments on the experiments performed and the obtained results in [5], these are:

1. The comparison between the proposed scheme and the reference scheme was only done for the case where the replenishment process assumes to be general. In the general case the proposed solution technique, DualNet, out performed the reference scheme that assumed independent identically distributed (i.i.d) replenishment process. However no comparison experiments were made to show the performance of both schemes if the replenishment process can be modeled as an i.i.d process. Therefore we have no idea which scheme would be better for such a replenishment profile.
2. A time slot duration of one minute was used for the numerical experiments in [5]. We believe that this is impractically too large (in practical TDMA systems time slots are in the order of milliseconds). Moreover, it takes more than five thousand time-slots (i.e., 5000 min) for the lower bound to be the closest to the upper bound as shown in Figure 16. This is too impractical in our opinion.
3. Even after the lower bound becomes closest to the upper bound (after a long time), it keeps decreasing monotonically again over ten thousand time slots (i.e., ten thousand minutes) by around 17% before starting to increase again.

## 11. Routing in Multi-hop Single Immobile Sink for Different Objectives under Distance Uncertainties

Optimization models were considered in [6] for WSNs subject to distance uncertainty for three different objectives:

- minimizing the energy consumed,
- maximizing the data extracted,
- maximizing the network lifetime.

Robust optimization was used to take into account the uncertainty present. In a robust optimization model the uncertainty is represented by considering that the uncertain parameters belong to a bounded, convex uncertainty set. A robust solution is the one with best worst case objective over this set.

### 11.1. Problem Statement and Design Objectives

For the three different types of problems, energy consumption was considered. The transmission and reception energy for each node is accounted for after normalizing with respect to the radio energy dissipation of the transmitter and receiver circuits. The expression for the total normalized energy has two components:

- one for the normalized received energy which is equivalent to the number of received bytes, i.e.,
  (24) $$\sum_{j|\left(j,i\right)\in A}f_{ji}$$
- and one for the the normalized transmitted energy, which is equivalent to the number of transmitted bytes times a linear function in the transmission distance, i.e.,
  (25) $$\sum_{j|\left(j,i\right)\in A}f_{ji}\left(1+\beta d_{ij}^{2}\right)$$

where

- *A* is the set of nodes in the network,
- $f_{ij}$ is the number of transmitted bytes from node *j* to node *i*.
- $d_{ij}$ is the distance from node *i* to node *j* and β is a constant depending on transceiver parameters.

### 11.2. Formulations for the Three Problems

A brief description of each of the three different optimization problems that were given in [6] is as follows:

1. *The Minimum Energy Problem*:
  - Decision variables: are the continuous variables $f_{ij}$ which are the number of bytes transmitted on a link from node *i* to node *j*.
  - Objective function: is a continuous linear objective function in $f_{ij}$ which is the sum of the transmission and reception normalized energies of all nodes in the network. The objective is to minimize that aggregate energy function.
  - Constraints: the first constraint is a minimum data transmission requirement constraint that requires the aggregate data transmitted from all nodes to the sink node, n+1 to be greater than a minimum number of bytes. The second and third constraint sets are conservation of flow constraints that require the difference between the amount of data bytes transmitted and received by a node to be less than the available data bytes at the node and greater than zero. The variables have non-zero constraints also.
2. *The Maximum Data Extraction Problem*:
  - Decision variables: are $f_{ij}$ which are the number of bytes transmitted on a link from node *i* to node *j*.
  - Objective function: maximizes the data transmitted to the sink node. It was given as the sum of data bytes $f_{ij}$ transmitted from each node *i* to the sink node n+1 on the arcs $\left(i,n+1\right)$. It is a continuous linear objective function.
  - Constraints: Besides the conservation of flow and non-negativity constraints in *Minimum Energy Problem*, there is a set of energy limitation constraints for each node, that guarantees that the the sum of transmitted and received energy for each node does not exceed the available energy of the node, i.e.,
    (26) $$\sum_{j|\left(i,j\right)\in A}f_{ij}\left(1+\beta d_{ij}^{2}\right)+\sum_{j|\left(i,j\right)\in A}f_{ij}\leq E^{i}\forall i\in N.$$
    Note that in [6], the energy is normalized such that $E^{i}$ is the number of bytes that could be transmitted with the available energy and the left hand side of the constraint is the amount of bytes transmitted for an expended amount of energy. All constraints are linear and hence the problem is a linear program ignoring the uncertainties.
3. *Maximum Lifetime Problem*:
  - Decision variables: are $f_{ij}$ which are the number of bytes transmitted on a link from node *i* to node *j* and *T* which is the variable represents the lifetime of the network.
  - Objective function: maximize the lifetime *T* of the network which is defined as the lifetime of the first sensor whose battery gets depleted, i.e., $T=min\left\{T_{1},T_{2},\ldots ,T_{n}\right\}$.
  - Constraints: Conservation of flow and non-negativity constraints typical to those in *Minimum Energy Problem*, in addition a quadratic constraint with bilinear terms that guarantees that the energy expended by transmission of a node *i* does not exceed its available energy, this is given by:
    (27) $$T\left(\sum_{j|\left(j,i\right)\in A}f_{ji}+\sum_{j|\left(j,i\right)\in A}f_{ji}\left(1+\beta d_{ij}^{2}\right)\right)\leq E^{i}$$
    where the left hand side This gives a quadratically constrained program, which is transformed to a linear program by substituting the variable *T* in the problem with q=1/T and minimizing the objective function instead of maximizing it.

### 11.3. Accounting for Uncertainties in the Formulation

According to [6] a robust solution for an optimization problem under uncertainty is defined as the solution that has the best objective value in its worst case uncertainty scenario. For an optimization problem under uncertainty with decision vector *x* and uncertainty vector parameter *u*, the robust solution is defined as:

(28) $$\underset{x}{min}\left\{\underset{u}{max}\;f\left(x,u\right):g\left(x,u\right)\leq 0\;\forall u\in U\right\}$$

which is equivalent to:

(29) $$\underset{x,\gamma }{min}\;\gamma :g\left(x,u\right)\leq 0,f\left(x,u\right)\leq \gamma \;\forall u\in U$$

where U is a closed convex uncertainty set. According to [6], the complexity of solving the robust counterpart of an optimization problem is equivalent to solving the deterministic problem for many problems. Moreover the increase in size of the problem is polynomial in the deterministic problem dimensions.

For the three optimization problems, the distance parameter was considered as the uncertainty parameter and hence in [6] was made to belong to the uncertainty set U. The set U defines distance vectors that are within a certain distance from a given estimate of the distance vector between nodes. The paper presented two general convex sets for the uncertainty region, these are polyhedral sets and ellipsoidal sets. For both types of uncertainty sets, the three deterministic optimization problems that were considered were formulated to their robust counterparts. The type of optimization problems obtained for the three cases are as follows:

1. Polyhedral uncertainty sets: Using LP duality, it was shown that optimization problem remains as a linear program with additional variables and constraints.
2. Ellipsoidal sets: Using a known closed form solution for an embedded ellipsoidal optimization subproblem with respect to the uncertainty variables, the robust optimization problem becomes a conic convex problem that can be solved by interior point methods in polynomial time. With a simple reformulation trick of replacing the conic component in the objective function with an upper bound linear component and bringing in the conic component in the constraint set, the problem can be rewritten as a second order cone program (SOCP).

### 11.4. Important Results

**Figure 17:**
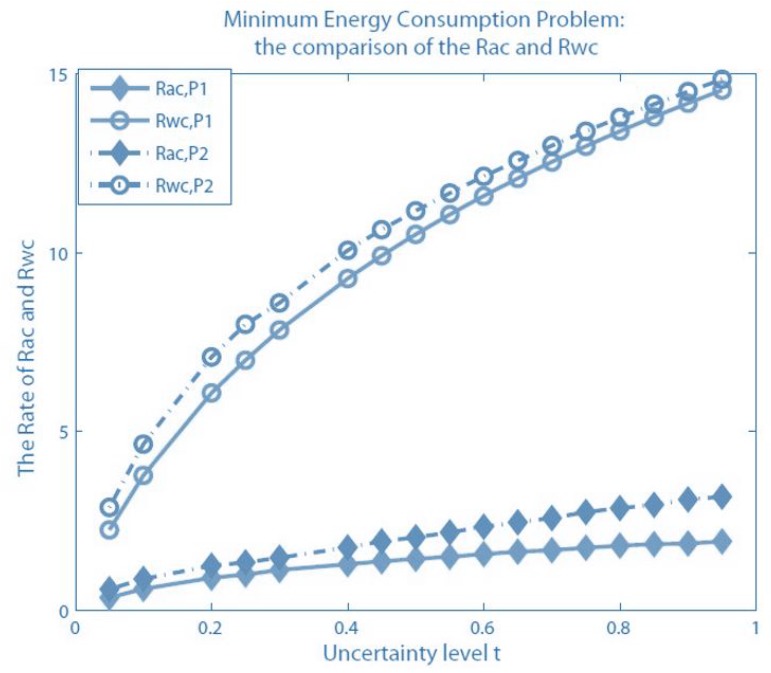
Ratios $R^{ac}$ and $R^{wc}$ for the minimum energy consumption problem versus the uncertainty levels [6].

The experimental results in [6] showed the relative loss of optimality on the nominal distance measurements ($R^{ac}$) and the relative increase of the deterministic solution in the worst uncertainty case ($R^{wc}$) versus the uncertainty level for all three problems mentioned earlier in this section. It was observed that $R^{wc}$ was greater than $R^{ac}$ with the difference increasing versus the uncertainty level, indicating that robust solutions are more attractive at the expense of small performance losses. This is illustrated in Figure 17 for different minimum data requirements to be extracted to the sink node ($p_{1}$ and $p_{2}$).

**Figure 18:**
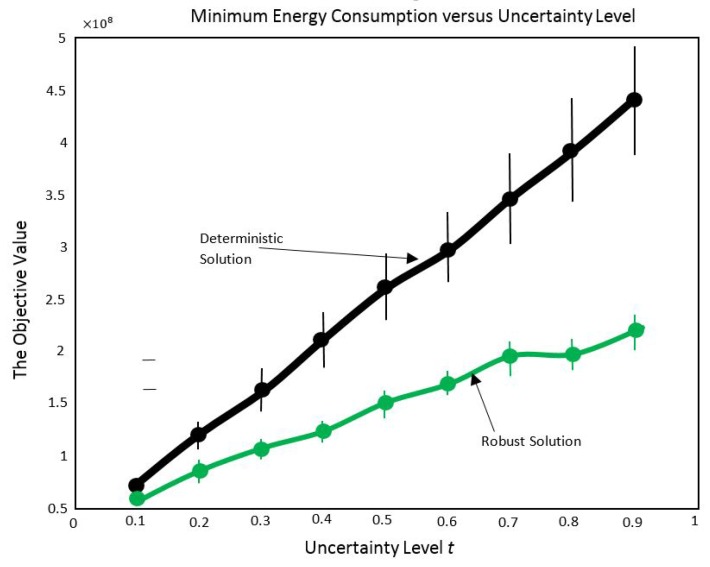
Mean and standard deviation of objective value for deterministic and robust solutions in minimum energy consumption problem for different uncertainty levels [6].

Furthermore, simulation results showing the obtained objective function values for the robust and deterministic solutions were also provided in [6]. The robust solutions were obtained for different degrees of uncertainty, while the deterministic solutions assumed complete certainty. The results showed the average and standard deviations of the objective functions over 100 random experiments. The deterministic solution gave a higher average objective function value compared to the robust solution while the standard deviation of the uncertainty level of the robust objective value was closer to its mean values as compared to the deterministic objective values. This is clearly illustrated in Figure 18.

## 12. Joint Routing and Scheduling in WSNs with Multiple Sinks having Different Location Possibilities

### 12.1. System Model and Design Objectives

In [7] scheduling and routing of data to multiple sinks having multiple position possibilities were considered. The objective is to maximize the network lifetime which is defined in [7] as the time elapsed since the launch of the network till the instant a living node cannot find a route to send its data to the sinks due to many dead nodes. The authors propose two formulations for the same problem which they state that they are equivalent but do not provide a proof for that.

### 12.2. Initial Problem Formulation: Time Based Formulation

The first formulation considered the time to be continuous and an independent variable based upon which all decision variables are dependent. It was named as *time-based formulation* and was considered very hard to tackle. A brief description of this formulation is as follows:

- Decision variables: *T* is a continuous variable that represents the network’s lifetime, $g_{ij}\left(t\right)$ is a continuous variable that represents the data rate on the link from node *i* to node *j* at a given time *t*, $g_{io}\left(t\right)$ is a continuous variable that represents the data rate from node *i* to one of the possible sinks’ positions *o* at a given time *t*, $x_{s,o}\left(t\right)$ is a binary variable that is set only when sink *s* resides in position *o*.
- Objective function: is to maximize the lifetime of the network which is given as $\underset{T,g_{ij}\left(t\right),g_{io}\left(t\right),x_{so}\left(t\right)}{max}\;T$,
- Constraint Sets: The constraints sets of the initial formulation are explained below:
  1. Constraint set 1: is a linear constraint set in the binary variables $x_{s,o}\left(t\right)$ that guarantees that a possible position *o* at *t* can get occupied by no more than one sink node, this is given as $\sum_{s\in V_{s}}x_{s,o}\left(t\right)\leq 1,\;\;\forall o\in V_{o}$, where $V_{s}$ and $V_{o}$ are the sets of sink nodes and possible sink positions respectively.
  2. Constraint set 2: is a linear constraint set in the binary variables $x_{s,o}\left(t\right)$ that guarantees that sink *s* at time *t* can only reside in one location, this is given as $\sum_{o\in V_{o}}x_{s,o}\left(t\right)\leq 1,\;\;\forall s\in V_{s}$.
  3. Constraint set 3: linear conservation of flow equality constraints in the flow variables $g_{i,j}\left(t\right)$ and $g_{i,o}\left(t\right)$.
  4. Constraint set 4: variable upper bounds on the flow variables $g_{i,j}\left(t\right)$ from node to node links that represent the link capacity.
  5. Constraint set 5: a mixed integer linear constraint in the variables $g_{i,o}\left(t\right)$ and $x_{s,o}\left(t\right)$ that impose link capacity on the flows from node *i* to the possible sink location *o* if any sink is assigned to that location, otherwise the flow is enforced to be zero. The constraint is $g_{i,o}\left(t\right)\leq C_{i,o}.\sum_{s\in V_{s}}x_{s,o}\left(t\right)\forall l_{i,o}$ where $C_{i,o}$ is the capacity of the link $l_{i,o}$.
  6. Constraint set 6: An energy constraint for each sensor i∈S which is an integration of linear terms with respect to the time parameter *t* with the decision variable *T* in the upper limit of the integral.
  7. Constraint set 7: non-negativity constraints on all the decision variables.

### 12.3. Reformulation: Pattern Based Formulation

As was mentioned for the time-based formulation in [7], the life-time variable *T* is connected to the rest of the variables through the energy constraint by integrating over time. This constraint complicates the problem and makes it difficult to solve according to [7]. Therefore a reformulation was performed to obtain an easier problem which discretized the time parameter into different durations to give an easier problem which has no integration in any of the constraints. For each duration, a placement pattern can be assigned such that the amount of energy expended over all time durations is within the initial available energy level of each node. The life time of the network is hence equivalent to the aggregate discretized time durations. In each time duration, an assigned pattern should satisfy all the constraint sets that were explained for the time-based formulation. The energy constraint in a given time duration $t_{p}$ for placement pattern *p* becomes a linear constraint in the flow variables $g_{i,j}^{p}$ and $g_{i,o}^{p}$. The elements of the reformulated problem are [7]:

- Decision variables:
  1. the continuous variables $t_{p}$ which represent the assigned time durations for the possible patterns *p*,
  2. continuous variables $e_{i}^{p}$ for the energy consumption rate for node *i* in pattern *p* for all nodes and patterns,
  3. binary decision variables $x_{s,o}^{p}$ tell whether sink node *s* is assigned to location *o* in the pattern placement *p* for all nodes, sinks and patterns,
  4. continuous variables $g_{i,o}^{p}$ for the data rate flow from node *i* to the sink position *o* in pattern *p* for all nodes, sinks and patterns,
  5. continuous variables $g_{i,j}^{p}$ for the data flow rate on the link between the nodes *i* and *j* in pattern *p* for all nodes and patterns.
- Objective function: Maximizes the aggregate durations assigned to the patterns which in [7], was stated to be equivalent to the lifetime of the network, i.e., $max\;\;T=\sum_{p\in P}t_{p}$.
- Constraint sets: The same constraint sets for the time-based formulation should be satisfied for each pattern, the energy constraints however are replaced by two constraint sets for each pattern, one is linear and the other is bilinear quadratic. The linear one is an equality constraint that links the energy expended by the node in a given placement pattern with the flow variables $g_{i,j}^{p}$ and $g_{i,o}^{p}$. The bilinear constraint set guarantees that all the energies expended by every node in all pattern durations do not exceed their initial battery energies.

### 12.4. Solution Method: Column Generation Method

Since the possible patterns are too large to enumerate, the column generation method was used in [7] for solving the following master problem obtained from the pattern-based formulation in [7]:

(30) $$\underset{t_{p},e_{i}^{p}}{max}\;T=\sum_{p\in P}t_{p}:\;\sum_{p\in P}e_{i}^{p}t_{p}\leq E_{i}\;\;\forall i\in V,\;\;e_{i}^{p},t_{p}\geq 0\;\forall \;i,p$$

where $V_{s}$ is the set of sink nodes, $V_{o}$ is the set of sink possible locations and *V* is the set of non-sink sensor nodes.

Different patterns correspond to columns in [7]. The master problem is given by the pattern-based formulation except for $e_{i}^{p}$ which is treated as a constant. It is hence a linear programming problem. The subproblem that determines which column to enter solves for the pattern that would give the maximum increase in the objective function of the master problem. If the objective function of the master problem cannot be improved any further then the optimal solution has been reached.

An initial set of patterns $P_{0}$ can be obtained by random assignment of sinks to locations and using shortest path Dijkstra’s algorithm for routing to the nearest sink. The master problem is then solved and the corresponding optimal dual variables are obtained to substitute in the objective function of the sub-problem, which is given by a linear equation representing the reduced cost of the master problem. The reduced cost is function in $e_{i}^{p}$, which is the only variable in the objective function of the subproblem. The constraint sets for the subproblems are linear conservation of flow constraints and flow capacity constraints on all links including the links to possible sink locations.

### 12.5. Important Results

For a network of four sensor non-sink nodes, one sink node and two possible locations for the sink, the problem instance was solved to optimality in less than one second taking less than six column generation iterations. For larger problems the computational time is unknown according to the authors in [7]. The experiments also showed that the mobility of sinks across possible locations can dramatically extend the network lifetime.

## 13. Delay-Sensitive Routing in Underwater WSNs

### 13.1. System Model and Design Objectives

Underwater acoustic WSNs (UWA-SNs) were considered in [8]. The propagation delay in UWA-SNs is five times larger than in RF networks which has a non-negligible impact on the performance of UWA-SNs especially since they cover much larger areas (square kilometers) unlike the RF WSNs. In [8] the propagation delay sensitivity was accounted for in optimizing energy for routing purposes. According to the authors of [8], this was not considered before their work due to the added complexity it brings in.

The sensor nodes are immobile and have relaying capabilities that enables the WSN to use multi-hop communication to convey a packet from any sensor to a sink node. Slotted synchronous time division multiple access (TDMA) was the type of medium access control (MAC) scheme considered. The objective was to design an offline routing scheme to pre-calculate the number of slots per link and the amount of data to be transmitted on each link while factoring in the large propagation delays of water acoustic signals. The routing scheme proposed in [8] was named Delay-constrained Energy-constrained Routing (DER).

### 13.2. Initial Problem Formulation

The initial problem formulation in [8] is a *nonlinear program* (NLP) with a non-deterministic generic objective function in the decision variables and linear sets of constraints. A brief explanation of the formulation is given as follows:

- Decision variables:
  1. the number of bits transmitted on each link ($W_{ij}$),
  2. the transmission time allocated for each link ($\Delta_{ij}$).
- Objective function: is the sum of all energy consumed on all links in the network. It contains $P\left(\frac{W_{ij}}{\Delta_{ij}}\right)$ which is a non-deterministic general term that is function in the number of bits $W_{ij}$ to be transmitted on each link and the allocated transmit time $\Delta_{ij}$. This term represents power consumption which is dependent on the modulation/channel coding schemes used for transmission.
- Constraint sets:
  1. A linear delay constraint, that takes into account the propagation delay of each link and the transmission delay that is inherent in the allocated transmission time. The total delays on all links from the source sensor nodes to the sink should not exceed a maximum allowable delay *T*, that was given by $\sum_{i=1}^{N-1}\sum_{j\in \psi_{i}}\left(\Delta_{ij}+\tau_{ij}\right)\leq T$ where $\tau_{ij}$ is the propagation delay of link $\left(i,j\right)$ and $\psi_{i}$ is the set of next hop candidates that are closer to the sink than *i*.
  2. A linear conservation of flow set of constraints for every node that guarantees that difference between incoming and outgoing data is equal to the amount of data generated by the sensor node, that is $\sum_{j\in \psi_{i}}W_{ij}-\sum_{j\in \psi_{i}^{{}^{\prime }}}W_{ij}=r_{i}T$ where $r_{i}$ is the data generation rate of node *i* and $\psi_{i}^{{}^{\prime }}$ is the set of neighboring nodes for which *i* is a next hop candidate.

### 13.3. Comments on the Initial Problem Formulation

It is worth noting that upper and lower bounds on the decision variables were not enforced in the main formulation. We believe that the number of bits per link should be lower bounded by zero and upper bounded by the link’s capacity. The transmission time should be lower bounded by zero too.

### 13.4. Reformulation

The objective function is linearized by substituting $P\left(\frac{W_{ij}}{\Delta_{ij}}\right)\Delta_{ij}$, the non-linear term, with an auxiliary variable $\epsilon_{ij}$ and adding to the constraint set the following equality constraint:

(31) $$log\left(\epsilon_{ij}\right)=P_{L}log\left(W_{ij}-log\left(\Delta_{ij}\right)\right)+log\left(\Delta_{ij}\right)$$

as well as the following equality constraint that connects the logarithm of the transmission rate with the number of bits transmitted and the transmission time allocated:

(32) $$log\left(R_{ij}\right)=log\left(W_{ij}\right)-log\left(\Delta_{ij}\right)$$

where

- $\epsilon_{ij}$ is a new variable that represents the energy expended for transmission of data on link $\left(i,j\right)$.
- $P_{L}$ is a constant that represents the relation between the logarithm of the power on a particular link with respect to the logarithm of the transmission rate on that link.

The non-linear inequality constraints (31) and (32) are piecewise linearized by Special Ordered Set type 2 (SOS2) variables and break points giving a *Mixed Integer Linear Program* (MILP). To approximate the logarithms of the energy, power and the allocated time on each link, five vectors of *k* SOS2 variables were introduced where *k* is such that the approximation error is within 1%. Not more than two adjacent SOS2 variables can be non-zero otherwise the rest of the variables are enforced to zeros.

### 13.5. Comments on Reformulation

The reformulation obtained has the following merits and demerits:

**Advantages**: Enabled elimination of the undetermined non-linear general objective function for which the problem had no known solution procedure. The resultant problem is an MILP which has deterministic techniques to solve.
**Disadvantages**: The accuracy of the approximation using the SOS2 variables is dependent on their number. Therefore for better solution approximation, a large number is needed.

### 13.6. The Solution Method in and Our Comments on it

There was no mention for the solution algorithm to be deployed by the network. A generic MILP solver is believed to be used for the numerical experiments, however it was not stated which procedures of those should be implemented in the network entity responsible for the routing implementation. Moreover the solution is to be implemented in a centralized fashion, which means lots of communication overhead is needed to update the nodes of the bit-load and transmission time. A high communication overhead in a very slow propagation system, like underwater acoustic system, is expected to greatly increase the time for which each node receives its routing information which leads to slow convergence.

## 14. Using Mobile Radio Frequency (RF) Power Charger to Charge the Batteries of Sensors in a WSN

### 14.1. System Model and Design Objectives

In [9], a WSN was considered in a smart grid with immobile nodes that utilize radio frequency (RF) energy of a mobile charger that visits specific landmarks to replenish their batteries. This was an extension to previous work (the scheme SuReSensE) that minimized the number of landmarks by considering priorities of the monitored equipment in the grid. The proposed scheme in [9], *Differentiated RF Power Transmission* (DRIFT), aims to deliver more power to high priority nodes using a mobile power transmitter by solving an integer linear program (ILP) to determine its optimal landmark positions. The possible landmarks of the mobile charger were assumed to be known in advance.

### 14.2. Problem Formulation

A brief explanation of the formulation is given as follows:

- Decision variables:
  1. $l_{xy}$, are binary variables indicating whether a landmark is assigned for the charger at the position (x,y) and
  2. $z_{xy}^{i}$, are binary variable indicating whether sensor *i* receives power from landmark (x,y).
- Objective function: is a binary integer linear function that maximizes the total received power of high priority nodes by selecting suitable land marks to be visited by the mobile wireless power charger and is given by $\sum_{i}\sum_{x}\sum_{y}\beta^{i}P_{xy}^{i}l_{xy}$ where $\beta^{i}$ is a binary constant whose value is 1 if the sensor node *i* is collecting data from critical equipment and hence is high priority and $P_{xy}^{i}$ is the power received by sensor *i* from a landmark positioned at (x,y) coordinates which depends on the pre-known euclidean distance.
- Constraint sets: are all binary integer linear constraints and their interpretations are explained below:
  1. A linear constraint set in the binary variables $l_{xy}$ that ensures that the total number of landmarks assigned to the charger do not exceed a certain pre-selected number.
  2. A linear constraint in the variables $l_{xy}$ and $z_{xy}^{i}$ enforces that if a landmark at a position (x,y) is chosen, then there has to be at least one sensor that could recharge its batteries from a mobile wireless charger at that position.
  3. A linear constraint in $z_{xy}^{i}$ that ensures that the power supply of the mobile charger is not exceeded, given the replenishment demand $\delta_{i}$ for node *i*:
    (33) $$\sum_{i}z_{xy}^{i}\delta_{i}\leq \tau_{l}\;\;\forall x,y$$
    where $\tau_{l}$ is the initial supply of the mobile wireless power transmitter.
  4. A linear constraint set in both $z_{xy}^{i}$ and $l_{xy}$ to ensure that a sensor can receive power from the position (x,y) only when there is a landmark for the charger in that position.
  5. A linear constraint in both $z_{xy}^{i}$ and $l_{xy}$ guarantees that each sensor is receiving power from at least one landmark.
  6. A linear constraint set in $z_{xy}^{i}$ ensures that sensor *i* receives power from a landmark in the position (x,y) only if it is within the transmission range of the mobile charger.
  7. A linear constraint set in $z_{xy}^{i}$ to ensure that the high priority nodes receive power that is at least equal to what the lower priority nodes receive.

### 14.3. Solution Method

From the context of [9], the solution of the problem is to be implemented in a centralized fashion. There were no specific algorithmic procedures mentioned. CPLEX solver was used to solve the problem.

### 14.4. Observations and Comments on the Problem Formulation, Solution Method and Experiments

The following are our comments on on the problem formulation in [9]

1. We believe that the constraint given in Equation (33) is not accurate because it accounts only for the energy expended in one location. A mobile charger can visit different landmarks, therefore, to guarantee that its total supply is not violated, then the summation should be done over all the possible landmark positions (x,y), i.e.,
  (34) $$\sum_{x}\sum_{y}\sum_{i}z_{xy}^{i}\delta_{i}\leq \tau_{l}$$
  This will also give an advantage of reducing the number of constraints for this set from x×y constraints to only one constraint.
2. The algorithm type and its details that solves the ILP was not mentioned. Choosing a suitable algorithm for e.g., branch and bound (BnB), and experimenting different branching methods, different relaxations to bound the subproblems for each node in the BnB tree, different methods of node selection and maybe integrating cutting planes (that could be problem specific) can greatly reduce the storage requirements and computational effort.
3. The positions of the land marks (x,y) are discrete in this formulation, however in practice they are continuous. The discretization could be an acceptable technique but the resolution definitely has an impact on the solution and the required computational effort. A high resolution gives the most accurate solution but leads to a large problem size requiring high storage and computational effort. Low resolution is vice versa.
4. The results illustrated in [9] compared the system performance mainly in terms of received power by the sensor nodes for DRIFT and the older proposed reference scheme SuReSense. No numerical results were provided to show the required computational effort and/or time of the proposed DRIFT ILP program and no information regarding the discretization of the landmark position was provided.
  We believe that some numerical experiments need to be done for different resolutions and the most suitable resolution in terms of the objective function value and the resulting problems size could be selected. For a mobile wireless charger, it is expected that the storage and computational capabilities to be very limited and hence should be taken into consideration when selecting a particular resolution.
5. It is expected for large WSNs, to have more than one mobile wireless charger, therefore we believe that it would be useful if the problem is extended to propose a distributed algorithm that balances the computational effort of solving the problem across the different mobile wireless chargers.

## 15. Assignment of Processing Tasks Across Nodes in a WSN

### 15.1. System Model and Design Objectives

In [10], the problem of finding the optimal deployment of tasks of a single operation across the nodes in a WSN with a single immobile sink was considered. Processing task distribution enables the utilization of the processing capability of all the nodes in the network to do the computations required for different applications before they arrive to the sink for further processing. Processing tasks across the nodes in a WSN consume processing energy. Therefore, due to the limited energy of irreplaceable sensor batteries, the objective is to find the combination of task assignment across the nodes to maximize the lifetime of the network. The network lifetime was defined in [10] as the duration before the first node fails due to battery exhaustion.

### 15.2. Initial Optimization Problem Formulation

A max-min problem that maximizes the minimum lifetime across all nodes was initially formulated as a binary non-linear program. A brief explanation of the formulation is given as follows:

- Decision variables: are given by a binary state vector $s_{i}\in {\left\{0,1\right\}}^{L}$ which represents assignment of the *L* processing tasks for an operation for each node *i*.
- Objective function: is a max-min nonlinear function in the binary decision variables given by the equation,
  (35) $$\underset{s_{i}\;\forall i\in X}{max}\underset{i\in X}{min}\frac{\gamma_{i}}{E_{i}\left(S\right)}$$
  where $\gamma_{i}$ is the initial battery residuary energy for node *i*, *X* is the set of nodes and $E_{i}\left(S\right)$ is the energy expended by node *i* for task processing and packet radio transmission. $E_{i}\left(S\right)$ is linearly dependent on the binary matrix S which is a concatenation of all the state vectors of all nodes $s_{i}\in {\left\{0,1\right\}}^{L}$.
- Constraint sets: the only constraint set aside from the binary nature of the decision variables is an upper bound binary constraint, $d_{i}\geq s_{i}\;\;\forall i\in X$, which ensures that no task gets assigned to a node except if it is allowed to (or capable to) do it by enforcing a zero value for any variable $s_{il}$ if the corresponding $d_{il}$ is zero.

This problem is easily converted to a mixed binary integer program by replacing the term $\underset{i\in X}{min}\frac{\gamma_{i}}{E_{i}\left(S\right)}$ in the objective function by an auxiliary variable α and minimizing it instead while adding the linear constraint $\frac{E_{i}\left(S\right)}{\gamma_{i}}\geq \alpha$ to the constraint set. This problem is centralized and has a complexity that scales with both the number of nodes and the number of tasks.

### 15.3. Solution Method

To reduce the communication overhead that is encountered in centralized schemes, a heuristic iterative decentralized algorithm that relies on gossiping communications is used to solve a smaller mixed binary linear program (MBLP) for a given node and its inflow-neighbors. The MBLP complexity is hence not dependent on the size of the network, but is dependent on the density of the network.

### 15.4. Comments on the Solution Method

The procedures to be implemented by the nodes to solve their local MBLPs were not mentioned.

## 16. Hierarchical Clustering in a Heterogeneous Network

### 16.1. System Model and Design Objectives

Clustering in a network of heterogeneous nodes which contains both sensors and actuators was considered in [11]. Since the actuator nodes can be deployed with external power sources, they were chosen to be the cluster heads. The cluster head role is not passed to other nodes and therefore some general sensor nodes in the cluster could consume more energy to transmit directly to the cluster head than others. This leads to imbalance in the energy consumption in the clusters which would shorten the lifetime of the network. A multilevel hierarchical structure was proposed in [11] to solve this problem by selecting "intermediate nodes" that could relay the data from the general sensors in the clusters to the cluster head. The scheme assigns general nodes to intermediate nodes in order to minimize the maximum energy consumption across all the nodes in the cluster. The following are the main elements of the system model in [11]:

- A single cluster was considered in which there are three types of nodes, general (non-relaying) nodes, intermediate (relaying) nodes and a single cluster head node.
- A general node can either transmit to the cluster head directly (single hop) or via intermediate nodes (multihop).
- Each general node can forward data to only one intermediate node.
- The amount of packets that each intermediate node can receive and aggregate, $k_{i}$, depends on the remaining energy of intermediate node *i*.
- The problem was considered on a packet by packet basis. So in the model, each general node can transmit up to one packet to an intermediate node. Therefore, each intermediate node *i* can be assigned at most to $k_{i}$ general nodes.
- The node positions were fixed and known to each other.

### 16.2. Problem Formulation

A mixed binary linear program was formulated to select the intermediate nodes and assign to them other nodes to relay their sensed data such that the maximum energy consumption across all the nodes in the cluster is minimized. The formulation details are as follows:

- Decision variables: the two sets of decision variables are,
  1. $A_{u,w}$ are binary decision variables that determine whether a node *w* is used as an intermediate node to relay data from a general node *u*.
  2. $E_{max}$ is a continuous non-negative variable that represents the maximum consumed energy among all nodes in the cluster.
- Objective function: is a continuous single variable linear function that minimizes the maximum energy variable $E_{max}$.
- Constraint sets: the interpretation of the different constraint sets is given below,
  1. A linear constraint set in the binary variables $A_{u,w}$ that guarantees that the number of packets relayed by an intermediate packet do not exceed the maximum that it can receive from general nodes, i.e., $\sum_{u\in N_{u}}A_{u,w}\leq k_{w}$, where $N_{u}$ is the set of general nodes.
  2. A linear constraint set in the binary variables $A_{u,w}$ that ensures that one general sensor node can forward its packets to only one intermediate sensor nodes on a direct link, i.e., $\sum_{w\in N_{w}}A_{u,w}\leq 1$, where $N_{w}$ is the set of intermediate nodes.
  3. A constraint that contains both the continuous maximum energy variable $E_{max}$ and the binary assignment variables $A_{u,w}$. This constraint enforces an upper bound of the energies of all nodes and was given in [11] as $E_{max}\geq E_{i}\left(A\right)$. The energies of all nodes were shown in [11] to be linear in the assignment vector of the variables $A_{u,w}$.

### 16.3. Solution Method

The proposed scheme in [11] to solve the optimization problem is based on branch and bound framework combined with cutting planes.The proposed procedure yields a $\left(1+\epsilon \right)$ optimal solution to the MBLP problem. Lower (infeasible) bound is obtained by linear relaxation of the integer variables while upper (feasible) bounds are obtained in polynomial time by heuristic method named *lowest energy path searching algorithm* (LEPA) which is explained in detail in [11]. Cutting planes are added to reduce the gap between bounds until the reduction becomes minor. A subproblem of the branch and bound algorithm is removed (or pruned) when a factor $\left(1+\epsilon \right)$ of the lower bound exceeds the upper bound.

### 16.4. Comments on the Solution Method

There was no information on the convergence speed or time for the solution method provided in the results section. We believe it is is important to know how long does it take to solve the problem for different $\left(1+\epsilon \right)$ suboptimal solutions.

## 17. Energy Efficient Co-Operative Broadcasting at the Symbol Level

### 17.1. System Model and Design Objectives

In [12] symbol-level broadcasting in a WSN was considered where cooperation among nodes in which a node’s receiver utilizes the residual energy of weak signal emitted from distant nodes and accumulates those along with the aggregate signals from near-by nodes. This was referred to as *Cooperative Wireless Advantage* (CWA) in [12] where the connectivity of a node was defined by its ability to detect a symbol, either received directly from the source node or through relaying nodes, with sufficient signal to noise ratio (SNR) such that for a given signaling scheme, the bit error rate (BER) is acceptable. The objective is to find the optimal energy allocation such that the total energy across all nodes in the network is minimized while satisfying an acceptable BER or equivalently a certain SNR level.

The WSN considered in [12] has only one source node (node 1) broadcasting to all the other nodes in the network. Each node in the network, other than the source node, serves as both a destination and a relay for the transmitted symbol. The protocol proposed in [12] works at the physical layer by doing the relaying on a symbol by symbol basis.

The transmissions of each node are orthogonal to one another, which means the system is interference free. This means that the received energies from different relay nodes can be added to detect the transmitted symbol. Also, the transmission bandwidth was assumed large enough in so that all relayed versions of a symbol are resolvable. The firing order was also assumed to be the same as the receiving order at each node. Hence the authors in [12], Hong et al., stated that this simplified their problem to make the minimum energy solution dependent on the order of firing (transmission) among the nodes instead of the absolute firing time instants.

Moreover, the optimal firing order was assumed to be known to reduce the problem complexity, and the nodes were enumerated based upon the firing order such that $t_{f_{1}}<\ldots .<t_{f_{N-1}}$. The last node, *N*, does not need to make any transmission to contribute to the broadcasting since all the nodes that fired before node i=N have successfully received and relayed the pulse to the other nodes. The firing of node *i* increments the energy by $\gamma_{i,k}\left(\epsilon_{k}\right)=\epsilon_{k}{\left|A_{i,k}\right|}^{2}$ for all the nodes j>i, where $A_{i,k}$ is the channel gain between nodes *i* and *k* while $\epsilon_{k}$ is the transmission energy of node *k*. The SNR requirement of a node *k* is hence satisfied by the accumulation of $\gamma_{ik}\left(\epsilon_{i}\right)$ for i=1,…,k−1.

### 17.2. Optimization Problem Formulation

The formulated problem allows a centralized solution method only to solve it. The details of the optimization problem are as follows:

- Decision variables: There are only one set of variables $\epsilon_{i}$, which are non-negative continuous variables that represent the transmission energies of all nodes in the network.
- Objective function: Is a linear function in the energy variables of all the sensors. It represents the total transmission energy in the network and is given by $\underset{\epsilon }{min}\;\sum_{i=1}^{N-1}\epsilon_{i}$
- Constraint sets: There is one constraint set given by $\sum_{i=1}^{k-1}\gamma_{ik}\left(\epsilon_{i}\right)\geq 1\;for\;k=2,\ldots ,N$ which is a linear constraint set in the decision variables whose left hand sides form a lower triangular matrix.

### 17.3. Comments on the Optimization Problem Formulation

We have two comments on the LP formulation that the authors provided:

1. The limitations on energy availability for every individual node were not considered. At a particular instant, it is most likely that the remaining energy supply for each node can be different. Therefore, the solution for the problem could have an energy allocation for some of the nodes for which that nodes’ batteries may not be able to provide.
2. It is not clear, how the authors assume that the optimal firing order is known, since it is coupled with the optimal transmission energy level at each node. We believe that the firing order should be captured in the formulation they gave.

### 17.4. Optimal Solution Method and Our Related Comments

In [12] just a single sentence was given on how the formulated LP can be solved, which we quote:

“We note that the problem in (6) is a simple variant of the linear programming problem that can be solved simply with back-substitution starting from k = 2.”

Our understanding to this statement is that to get the minimum energy while satisfying the functional constraints, the solution has to be binding. This enables solving the set of equations using simple forward substitution since the coefficient constraint matrix for the constraint set is a lower triangular matrix. However if this is what was meant by the statement that we quoted, then it is not clear for us how, in a general network topology, will this solution method guarantee non-negative values for the decision variables.

### 17.5. Suboptimal Solution Method: A Heuristic Technique

Two heuristic algorithms were proposed that provides a feasible suboptimal solution to the problem, they were called *Cumulative Increment Algorithm* (CIA) and *Cumulative Sum Increment Algorithm* (CSIA). Both are iterative algorithms that update the energy assignment for each node in the network until the entire network is connected (i.e., satisfies its minimum required energy level for reliable detection).

For CIA, in each iteration, a pair of nodes, *j* from the set of connected nodes (TX) and *j* from the set of disconnected nodes (RX) are selected. The selection is such that the required increment in the transmission energy of the *j* is minimum. The transmission energy of node *j* is incremented by the amount of energy needed to bring the received energy phase level of node *i* to the required threshold.

In CSIA, the transmitter in TX that has the best aggregate link gains with the nodes in RX is chosen. This could make the energy phase for more nodes in RX reach their required threshold as compared to CIA. Therefore, according to [12] CSIA is saves more energy.

### 17.6. Important Numerical Results

Numerical solutions for the heuristic algorithms and the optimal solution were obtained for a 6 node network. The optimal solution was obtained using exhaustive search. The authors show that the solution obtained by their heuristic techniques is worse than the optimal by 5%. Furthermore, results for up to 140 nodes were obtained for the proposed heuristic algorithms, in terms of total network energy, versus two reference broadcasting schemes that do not utilize cooperation. It was shown that the proposed algorithms both outperform those that do not use cooperation with superiority to CSIA.

### 17.7. Comments on the Solution Methods and Their Related Experiments

1. The energy budget for every node was not considered in their heuristic algorithms. Therefore, there is no guarantee that the solution can always satisfy the available battery energy for every node.
2. The heuristic algorithms’ solutions were compared with that of the optimal solution for only six nodes. It is not clear how the gap between the heuristics and the optimal solutions behave for a large number of nodes.
3. The solution of the LP program formulated in [12] was not compared with the proposed heuristics. We believed it could be useful to compare its obtained solution with the heuristics and choose the one with the best performance as the LP formulation gives a suboptimal solution anyway.
4. The required computational effort or time for the heuristics was not stated. It is important to know this information in order to decide if the algorithms can be implemented in real time.

## 18. Dispatching of Mobile Sensor Nodes in a WSN to Sense a Region of Interest

### 18.1. System Model and Design Objectives

In [13], a sensing field *A*, an area of interest *I* inside *A*, and a set of mobile sensors *S* resident in *A* were considered. The dispatch problem considered finding a subset $S^{\prime }\subseteq S$ of the sensors to be moved to *I* such that the coverage and connectivity requirements are satisfied, and the movement energy cost is minimized. The positions where the set of sensors $S^{\prime }$ are to be located in *I* were obtained by the placements methods that were proposed in the same paper. Those placement methods are not mentioned here as they do not contain the optimization component we are interested in. Two solutions were proposed, a centralized one and a distributed one.

### 18.2. Problem Formulation

The problem was formulated as a maximum-weight maximum-matching problem. Two objective functions were considered, one minimizes the energy expended due to the motion of the sensors and one maximizes the average remaining energy of sensors after their movement. We find that both are equivalent to each other. The more distance traveled by a node, the more it expends energy for that motion.

Given a set of placement positions $L=\left\{\left(x_{1},y_{1}\right),\left(x_{2},y_{2}\right),\ldots ,\left(x_{m},y_{m}\right)\right\}$, the cost to move every sensor $s_{i}$ to a location $\left(x_{i},y_{i}\right)$ was calculated as $c\left(s_{i},\left(x_{j},y_{j}\right)\right)=\Delta_{m}d\left(s_{i},\left(x_{j},y_{j}\right)\right)$, where $\Delta_{m}$ is the energy cost per unit distance and $d\left(s_{i},\left(x_{j},y_{j}\right)\right)$ is the minimum distance between $s_{i}$ and $\left(x_{j},y_{j}\right)$. The weight of each edge is the negative of its cost.

The bipartite graph *G* that was used to model the assignment problem was balanced and transformed to $\hat{G}$ by adding the set of virtual location points $\hat{L}$ that act as dummy nodes of $\hat{G}$. $\hat{G}$ is hence a maximum-weight perfect-matching problem. The weights of the edges incident on virtual points are assigned a value lower than the weight of edges incident on non-virtual locations so that they have no impact on the solution.

**Figure 19:**
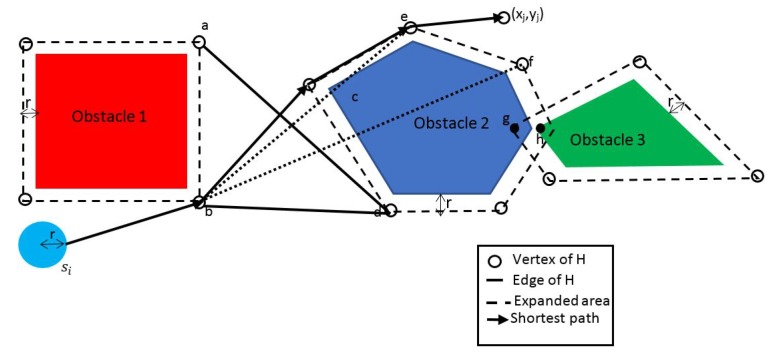
Finding a collision free path in [13].

The shortest distance $d_{i}\left(s_{i},\left(x_{j},y_{j}\right)\right)$ is supposed to be collision free on a path that can have obstacles as shown in Figure 19. To make sure that sensor $s_{i}$ does not collide with the areas’ boundaries or any obstacles, the sensor was modeled as a circle in a 2D plane with a radius *r*. The perimeters of the 2D obstacles were expanded by *r* and those of the boundaries are contracted by *r*. The positions of the corners of the expanded obstacles and contracted boundaries were made elements of the set of vertices *V*. The problem was then modeled as a graph $H=\left(s_{i}\cup \left(x_{j},y_{j}\right)\cup V,E\right)$ where *E* is the set of edges $\left(u,v\right)$ where $u,v\in \left\{s_{i}\cup L\cup V\right\}$ and the edge does not pass through any expanded perimeters.

### 18.3. Solution Method: Centralized

The shortest distance problem was solved using Dijkstra’s algorithm which is a greedy algorithm with quadratic complexity, while the Hungarian algorithm was used to solve the matching problem which has a cubic complexity.

### 18.4. Solution Method: Distributed

A distributed algorithm based on a greedy heuristic approach was proposed in [13] to obtain a suboptimal solution with lower communication and processing overhead. The algorithm requires periodically broadcasting a list of placement locations for the target area to the all sensor nodes in the network. The list identifies the locations that are occupied and those that are not. Initially all the locations are unoccupied and each node has a copy that may not be identical until convergence. Each node $s_{i}$ selects the shortest target location from the list to move to. While moving to its target, the sensor node broadcasts the status of its list of target locations and the cost to move to the destination. All locations marked as occupied by $s_{i}$ are also marked as occupied by the nodes receiving the list of $s_{i}$. If $s_{i}$ and $s_{k}$ are moving towards the same location they compete their costs and the one with the lower cost wins and keeps moving towards the destination while the other searches for the other nearest target position. Each sensor keeps repeating the process, while sending and receiving location targets and costs until either it reaches a destination or looses to the other sensors and finds that all locations are marked as occupied.

### 18.5. Comments on the Solution Methods

The following are our comments on the proposed solutions methods in [13]:

- The communication overhead required to periodically broadcast the location tables and the costs for each node seems to be high compared with the centralized case which only broadcasts the final result to all the nodes. A comparison was not provided for that issue in the results section. Generally, one of the main advantages for using distributed solutions over centralized is the reduced communication overhead in the exchange of information. It is not clear however whether this is satisfied in the distributed algorithm proposed in the paper.
- A comparison between the time required for the algorithms to converge is not provided. We believe that the centralized algorithm that was proposed in the paper converges fast compared to the distributed algorithm.
- The unnecessary energy expended by the sensor nodes when they compete and loose to others was not discussed in the results section.
- It is not clear, whether the distributed algorithm, that according to the published results consumes more energy due to the sub-optimality, has any advantages compared to the centralized algorithm.
- We believe that the broadcasting time interval setting is an important factor that affects the convergence time in the distributed algorithm. However, there were no results provided that shows that relation.

## 19. Fusion of Delay Sensitive Noise Perturbed Data Sensed by Different Nodes in a Given Cluster

### 19.1. System Model and Design Objectives

A cluster of a WSN was considered in [14] where sensor nodes collect and transmit data to the fusion center of the cluster directly or through relay nodes. Relay nodes amplify and forward data to the fusion center or the next relay node. Synchronous TDMA based MAC was considered in [14]. The total energy consumed by the sensor is related to the transmission power and the duration of transmission as the following equation shows:

(36) $$E_{k}=\left[\left(1+\alpha_{k}\right)P_{tk}+P_{ck}\right]T_{tk}+P_{0k}T_{0k}$$

where $P_{tk}$ is the data transmission power, $\alpha_{k}$ is the ratio of power consumption by RF power amplifier to transmission power, $P_{ck}$ is the circuit power in the transceiver baseband parts, $T_{tk}$ is the duration of the transmitting mode which is inversely proportional to the transmission rate, $P_{0k}$ and $T_{0k}$ are the power consumption and duration for the sleep and transient modes. The time delay per frame considered in [14] is the sum of transmission durations from the sensor nodes to the fusion center.

**Figure 20:**
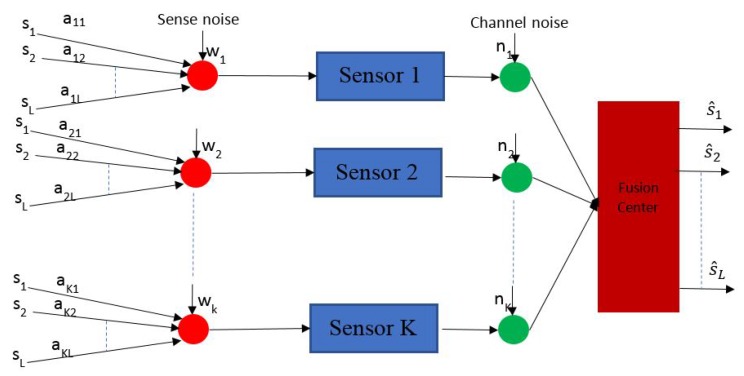
Data flow of noise perturbed data from the sensors in a cluster to the fusion center [14].

There are *L* physical phenomena sources to be observed in the given cluster by *K* sensors. The sensed data is in analogue format in each sensor which was considered as the weighted sum of all the original data being observed and the sensor’s noise which was assumed to be Gaussian. The analogue signal is quantized at the sensor and then digitized and sent on a noisy channel to the fusion center as shown in Figure 20. The fusion center uses the least square error method to estimate the original data from the received signals of the sensors with prior knowledge of the weighting coefficients. Since the battery life is limited and the data considered was delay sensitive, optimization of the transmission power allocation strategy and the delay of each node was considered to minimize the mean distortion of the measured information.

### 19.2. Optimization Problem Formulation

The details of the optimization problem are as follows:

- Decision variables: The sensor transmission powers and transmission durations, for all sensors in the nodes are the decision variables. These are all continuous variables.
- Objective function: is the mean distortion of the system.This is given as an undetermined function of the power vectors of the sensor variables. That is $\underset{P,T}{min}D\left(P\right)$. The relation between the objective function, the transmission powers and transmission durations in the system was not provided in [14].
- Constraints: The following are the constraint sets of the problem,
  1. A linear constraint in the transmission time duration variables to guarantee that sensed data arrive at the fusion center within the desired delay limits, that is $\sum_{k=1}T_{tk}-T_{T}\leq 0$
  2. A bilinear quadratic constraint in the transmission powers and delays that guarantees that the aggregate transmission energy does not exceed an allowable limit. That is $\sum_{k=1}^{K}E_{k}-E_{T}\leq 0$ where $E_{k}$ was given by Equation (36)
  3. Non-negative constraints on the transmission power values.

### 19.3. Comments on the Formulation

These are our comments regarding the problem formulation:

1. The variables $T_{k}$ should take non-negative values only. However, there is no non-negativity constraints for those variables in the formulation.
2. The relation of the objective function with the power decision variables of the sensor nodes and the transmission time duration decision variables of each sensor node is not provided.
3. In [14], it was mentioned that adaptive modulation is employed to adjust the transmission delay. However, this is not shown in the formulation. If adaptive modulation is used then $T_{tk}$ should only take finite discrete values depending on the modulation scheme selected. This would certainly affect the objective function, since it is dependent on the modulation scheme. The change will also affect the aggregate transmission energy constraint since $E_{k}$ is function in $T_{tk}$ as given in Equation (36).
4. The Lagrangian function and KKT conditions were obtained for the problem. The dual variables corresponding to the constraints were unrestricted, however the two dual variables should be restricted to be non-negative since they correspond to inequality constraints [38].
5. The equations obtained for KKT should also satisfy the primal functional constraints in the problem formulation [38]. The equations the authors obtained from KKT are not sufficient to satisfy feasibility.

### 19.4. Solution Method: Heuristic scheme

A heuristic technique was used to find modulation schemes for all the nodes that satisfy the delay constraint. The Lagrangian then becomes function only in the power decision variable vector P, and only one dual variable corresponding to constraint energy limit constraint. This gives a set of equations whose number is equal to the set of unknown variables. The Newton Method was used to solve the set of equations. Since KKT conditions are only necessary but not sufficient for a global solution, all the solutions for stationary points were obtained then the one that gave the minimum mean distortion was selected.

### 19.5. Comments on the Solution Methods

Our comments on the proposed solution methodology:

- The convergence of the Newton algorithm and the correctness of the convergence is highly dependent on the initial point and the type of functions in the set of equations. The nature of these functions is not clear since there was no explicit function for the mean distortion as function in P and T provided. Initial points that are not close enough to the solutions may lead to wrong convergence or no convergence at all.
- The Newton algorithm will usually converge to one solution given an initial point. In order to find the other solutions, then the algorithm should be invoked again with new suitable initial points. The problem is we do not know how many local solutions exist and how do the authors choose their initial points to correctly obtain all the solutions of the equations for the KKT conditions.
- Solving for all the stationary points can be computationally inefficient.
- The solutions for the set of equations are not guaranteed to have non-negative values for the primal and the dual variables.

## 20. Energy Optimization in Wireless Visual Sensor Networks While Maintaining Image Quality

In [15], Ghazalian et al. consider sensor nodes that are basically, camera nodes with different distances from the fusion center ( or sink). In their paper, they optimize energy consumption under the constraints of maintaining certain image quality by finding a camera node selection algorithm for target tracking applications, where energy consumption and image quality are connected.The field of view (FOV) and depth of the field (DOV) are the quality constraints considered in the optimization design problem in [15]. These constraints guarantee a desired image quality as well as an acceptable amount of coverage for the target being tracked. Ghazalian et al. also considered the number of quantizer bits required to represent an image pixel of the object being tracked, based upon the variance of the image.

### 20.1. System Model

Ghazalian et al. consider a network of camera sensors in [15] that do not involve any routing, but rather transmit directly to a fusion center. Each camera node consists of four modules that consume energy, these are the transmitter module, the compression module, the imaging module and the power module. Each sensor has $W_{s}^{i}$ pixels in horizontal direction and $H_{s}^{i}$ pixels in vertical direction. The average number of bits required to quantize each pixel value, after compression, is $N_{b}^{i}$ and the frame rate of imaging is assumed to be $f_{frame}$ fps (frame per second). The required bit rates for transmitting video from each camera node to the fusion center (the sink node) is then equal to $f_{frame}W_{s}H_{s}N_{b}$ bps (bits per second). To achieve a threshold SNR at the fusion center, a camera sensor node is required to transmit with the following power level:

(37) $$p_{i}^{t}=\frac{{\left(4\pi \right)}^{2}N\gamma_{th}B}{\lambda_{c}^{2}G_{r}G_{t}}{\left(d_{i}^{fc}\right)}^{2}$$

where *N* is the power spectral density of additive white Gaussian noise (AWGN), $\gamma_{th}$ is the SNR threshold, $\lambda_{c}$ is the carrier wavelength $G_{r}$ and $G_{t}$ are the receive and transmit antenna gains respectively, *B* is the transmission bandwidth and $d_{i}^{fc}$ is the distance between the wireless camera node *i* and the fusion center. The total amount of energy consumed by a camera sensor node is:

(38) $$E_{i}^{total}=p_{i}^{t}f_{frame}W_{s}^{i}H_{s}^{i}N_{b}^{i}+\alpha \Delta f_{i}+p_{i}^{cvid}T+\tau \Delta \theta_{i}$$

where $\alpha \Delta f_{i}$ is the amount of consumed energy by a miniature motor to change the focal length $f_{i}$ of the camera lens by $\Delta f_{i}$ using a force of α, and $p_{i}^{cvid}$ the power consumed by the imaging module for time *T*.

The *field of vision* (FOV) of a camera is the region where the camera can cover without any obstacles. It is given by:

(39) $$\phi =tan^{-1}\left(\frac{W_{s}}{2f_{i}}\right)$$

and must satisfy a minimum threshold $\phi_{th}$ that depends on the radius $radius^{target}$ of the target object as well as its distance $d_{i}^{target}$ from the camera sensor, given in [15] as:

(40) $$\phi_{th}=\frac{radius^{target}}{d_{i}^{target}}$$

The *depth of field* (DOF) is the difference between the distance of the front edge and the rear edge of a focused object. There is a far limit $Dep_{f}^{i}$ and a near limit $Dep_{n}^{i}$ that describe the DOF of node *i*. They depend on the diaphragm $Diaf_{i}$ of camera *i*’s lens, the focal length $f_{i}$ and the distance $d_{i}^{target}$ between the camera node *i*. They are given as:

(41a) $$Dep_{n}^{i}=\frac{d_{i}^{target}f_{i}^{2}Diaf_{i}}{Diaf_{i}f_{i}^{2}+cf_{i}\left(d_{i}^{target}-f_{i}\right)}$$

where *c* here is the circle of confusion diameter [39]. The input variance $\sigma_{th}^{2}$ of the capture target image decreases with the distance $d_{i}^{target}$. The authors were able to fit it acceptably by an exponential function given as:

(42) $$\sigma_{input}^{2}=e^{-0.06d_{i}^{target}}$$

and the variance $\sigma_{output}^{2}$ of the output image was derived in [15] as:

(43) $$\sigma_{output}^{2}=e^{-0.06d_{i}^{target}}\left(0.03N_{b_{i}}^{0.4}+0.91\right)$$

where $N_{b_{i}}$ is the number of bits per pixel that camera *i* uses for image quantization.

### 20.2. Formulation

Using algebraic operations on the equations of FOV and DOF, the authors of [15] derive a convex optimization problem. A brief explanation of the formulation is as follows:

- Decision variables:
  1. $\rho_{i}\in \left\{0,1\right\}$ selects decides on whether camera node *i* is selected to participate in the tracking process. The authors relax the binary constraint ($0\leq \rho_{i}\leq 1$) in the formulation and do not explicitly clarify how integrality is restored later.
  2. The number of encoding bits $N_{b_{i}}$ per pixel that camera *i* uses for image quantization. It is treated as a continuous variable in [15].
  3. the focal length $f_{i}$ of node *i*, which impacts the FOV and the DOF. It is a continuous variable.
- Objective function: Is the sum of the total energies, given in Equation (38), of each node $\sum_{i}^{N}\rho_{i}E_{i}^{total}$.
- Constraint sets:
  1. One camera node should be active in each scenario of target tracking, therefore, $\sum_{i}^{N}\rho_{i}=1$.
  2. Linear constraint sets in the $f_{i}\geq \rho_{i}F_{min}$.
  3. Bilinear constraint sets $f_{i}\rho_{i}\leq F_{max}$.
  4. The encoded image variance $\sigma_{out}^{2}$ should be greater than a threshold σth. This is represented by a nonlinear constraint in $N_{b_{i}}$ and $\rho_{i}$, given as:
    (44) $$\rho_{i}e^{-0.06d_{i}^{target}}\left(0.03N_{b_{i}}^{0.4}+0.91\right)\geq \rho_{i}\sigma_{th}^{2}$$

### 20.3. Solution

The Lagrangian function was obtained as:

(45) $$\begin{array}{c} L=\sum_{i}^{N}\rho_{i}E_{i}^{total}+\lambda_{1}\left(\sum_{i}^{N}\rho_{i}-1\right)+\sum_{i=1}^{N}\lambda_{2,i}\left(\geq \rho_{i}F_{min}-f_{i}\right)+\sum_{i=1}^{N}\lambda_{3,i}\left(\rho_{i}f_{i}=F_{max}\right)+\\ \sum_{i=1}^{N}\lambda_{4,i}\rho_{i}\left(\sigma_{th}^{2}-\sigma_{out}^{2}\right)-\sum_{i=1}^{N}\lambda_{5,i}\rho_{i}+\lambda_{6,i}\left(\rho_{i}-1\right) \end{array}$$

where $\lambda_{i}$ and $\lambda_{k,i}$ (k=2,3,…,6) are the Lagrange multipliers of the constraints. Ghazalian et al. applied KKT conditions explained in Section 5.2, to the problem and obtained closed form expressions for the Lagrange multipliers as well as the primal decision variables $N_{b_{i}}$, $f_{i}$ and $\rho_{i}$. They used these expressions to devise a node selection algorithm that satisfies the following simultaneously:

- A selected camera node has the shortest distance to the fusion center.
- The distance between the target and the selected camera node should cause the minimum lens movements and the minimum number of bits for each pixel value.
- A selected camera node needs the minimum energy for the camera direction setting.

## 21. Conclusions

This paper was divided mainly into two main parts. Part II presented a basic tutorial on optimization techniques specifically in LP and NLP. We focused on their respective basic concepts that are required when devising a solution algorithm. Linear network flow and MST problems received special attention in our discussion due to their suitability in modeling many WSN design problems and their highly efficient algorithms. Introduction to primal and dual decomposition techniques was also provided in part II, which could provide us with distributed optimal solution schemes. In Part III we explored and illustrated how different optimization techniques were used in solving different design aspects in WSNs. We explained the formulations that were done for these problems, classified them according to their types and pointed out any observable weaknesses. We also explained the solution techniques used and the experiments carried on to evaluate their performance. We also commented on some of the experimental procedures and comparisons between solution schemes to reflect our opinions that could also suggest points that could be taken into account when using optimization techniques for design in WSNs. We mentioned how distributed schemes are highly desirable in WSNs to reduce the communication overhead among nodes and balance the computational energy consumption across the nodes. Distributed computation is hence an important element that we included in our observations of the optimization techniques used.

## Figures and Tables

**Table 1:** Example of a simplex tableau.

										Basic Variable
Row 0	*z*	$-60x_{1}$	$-30x_{2}$	$-20x_{3}$					=0	z = 0
Row 1		$8x_{1}$	$6x_{2}$	$x_{3}$	$s_{1}$				=48	$s_{1}=48$
Row 2		$4x_{1}$	$2x_{2}$	$1.5x_{3}$		$s_{2}$			=20	$s_{2}=20$
Row 3		$2x_{1}$	$1.5x_{2}$	$0.5x_{3}$			$s_{3}$		=8	$s_{3}=8$
Row 4			$x_{2}$					$s_{4}$	=5	$s_{4}=5$

**Table 2:** Time complexity for Prim’s algorithm using different implementations.

Minimum Edge Weight Data Structure	Time Complexity
adjacency matrix, searching	$O\left({\left|V\right|}^{2}\right)$
binary heap and adjacency list	$O\left(\left|E\right|\left|log\left(V\right)\right|\right)$
Fibonacci heap and adjacency list	$O\left(\left|E\right|+|\left|log\left(V\right)\right|\right)$

**Table 3:** Summary of the covered design problems and the corresponding optimization techniques.

**Design Aspect**	**Classification of Initial Problem Formulation**	**Objective**	**Initial Problem Formulation: Centralized/Distributed?**	**Classification of Reformulated Problem**	**Solution Method**	**Implemented Solution Scheme for the Problem: Centralized/Distributed?**	**Nature of Solution**	**Convergence Speed/Complexity**
Energy and route allocation from multiple source to multiple destination in rechargeable WSNs [5]	Complicated Convex NLP	Maximize Aggregate node utilities	Centralized	Decomposed to two easier strictly convex subproblems by decoupling the time component	Dual decomposition +subgradient algorithm used in a heuristic scheme	Distributed	suboptimal in finite time but optimal in infinite time	Too slow, took 5000 min to reach the best suboptimal solution in the simulations done in [5]
Joint Routing and TDMA scheduling for delay sensitive underwater acoustic WSNs [8]	NLP with general undetermined nonlinear term in the objective function	Minimize the transmission energy	Centralized	Mixed Integer Linear Program	Not stated, an MILP solver is assumed to have been used	Centralized	E Global optimal to the MILP but is suboptimal to the original NLP problem	Depends on the desired approximation error. Expected to be fast for large approximation errors but slow for small approximation errors, hence there is a trade-off.
Routing for a WSN Multi-hop with Single Immobile Sink [2]	bilinear quadratically constrained program	Maximizes the shortest lifetime across all nodes	Centralized	reformulated to linear program reformulated to strictly convex quadratic program	Dual Decomposition + subgradient algorithm	Partially distributed for linear program reformulation Fully distributed for the strictly convex quadratic program reformulation	E Global Optimal	Partially distributed algorithm: around 600 subradient iterations to converge Fully distributed algorithm: around 3500 subgradient iterations to converge
Joint Routing and Scheduling in WSNs with Multiple Sinks and Different Sink Location Possibilities [7]	Non -Linear and non-deterministic (due to the integration function)	Maximize the network lifetime	Centralized	Non-Linear Quadratically constrained Program	Column Generation Method	Centralized	Global Optimal	Too quick for small problems (less than one second and 6 iterations) but unknown for large problems
Dispatching of Mobile Sensor Nodes in a WSN to Sense a Particular Region of Interest [13]	Maximum-weight maximum-matching problem	Minimize the total expended motion energy	Centralized	N/A	Hungarian and Dijkstra’s algorithms	Centralized	Global Optimal	Cubic polynomial time complexity
					A greedy problem specific heuristic	Distributed	Suboptimal	Not provided
Fusion of delay sensitive noise perturbed data sensed by different nodes in a given cluster [14]	Non-linear Program with non-determinisitc objective function	Minimize the mean distortion of the measured data by the sensors	Centralized	N/A	Heuristics+ Newton’s Method	N/A	Suboptimal	Was not provided
Co-operative Broadcasting at the Symbol Level [12]	Linear Program	Minimize the aggregate transmission energy	Centralized	N/A	Heuristcs	N/A	Suboptimal	Not provided
Joint routing, power and bandwidth allocation in FDMA WSNs [4]	Non-strictly convex non-linear constrained program	Minimize the aggregate power for all network links	Centralized	Convex non-linear global consensus problem	Augmented Lagrangian +Alternating direction method of multipliers	Distributed among nodes	Global optimal	Fast, converged in 21 iterations of the ADMM algorithm
				Strictly convex non-linear program (using proximal regularization)	Dual decomposition and projected subgradient algorithm	Distributed among nodes and decomposed in protocol layers	Global optimal	Slower than ADMM, converges in 133 iterations of the projected subgradient algorithm
Assignment of processing tasks across the WSN [10]	Mixed binary linear program	Maximize the life time of the network (defined as the lifetime of the first node to die of exhausted battery)	Centralized	Mixed binary linear program local to each node and its neighbors	Heuristic that relies on gossiping to solve a local BILP at each node	Distributed	Suboptimal	Was not shown, however is expected to be quick if the density of the network is low (even for a large network) since the size of the local MILP problem for each node depends on the number of neighbors it has.
Routing in a delay-tolerant WSN to a Single Mobile Sink [3]	Quadratically Constrained Program	Maximize the number of cycles made by mobile sink	Centralized	Linear program	Lagrangian relaxation +subgradient algorithm, greedy algorithm for fractional knapsack problems+ trivial heuristic for box constrained linear program	Decentralized algorithm	Good Suboptimal. Global Solution is also attainable in a long time	Good suboptimal in few iterations (100-200) and global after too many iterations.
Hierarchical Clustering in a heterogeneous Network [11]	Mixed Binary Linear Program	Minimizes the maximum expended energy among nodes	Centralized	N/A	Branch and cut framework	Centralized	Could be solved to the desired degree of (1+ ϵ) optimality.	Was not Shown
Maximize total received power by rechargeable sensor nodes in a smart grid from a mobile wireless charger [9]	Binary Linear Program	Maximizes the total received power of high priority nodes	Centralized	N/A	Not stated, CPLEX solver was used to solve the problem	Centralized	Global optimum	Was not shown. However is expected to be faster for low resolution discretization and vice versa
Account for uncertainties in the distance between sensor nodes [6]	Linear Program	Aggregate transmission and reception normalized energies	N/A	Polyhedral uncertainty set: yields Linear Program Ellipsoidal uncertainty set: yields a second order cone program (SOCP)	Interior point methods, CPLEX solver was used to solve the problem	N/A	Global optimum	Can be solved in polynomial time
	Linear Program	Maximize the transmitted data to the sink						
	Bilinear Constrained Program	Maximize the network lifetime						
